# An integrated RNAseq-^1^H NMR metabolomics approach to understand soybean primary metabolism regulation in response to Rhizoctonia foliar blight disease

**DOI:** 10.1186/s12870-017-1020-8

**Published:** 2017-04-27

**Authors:** Tanya R. Copley, Konstantinos A. Aliferis, Daniel J. Kliebenstein, Suha H. Jabaji

**Affiliations:** 1 Plant Science Department, McGill University, Ste-Anne-de-Bellevue, Quebec, H9X 3V9 Canada; 2 Department of Plant Science, Laboratory of Pesticide Science, Agricultural University of Athens, Iera Odos 75, 118 55 Athens, Greece; 3 Department of Plant Sciences, University of California, Davis, 95616 USA

**Keywords:** *Glycine max*, Bidirectional orthogonal projections to latent structures, Primary metabolism, *Rhizoctonia solani*, Transcriptomics

## Abstract

**Background:**

*Rhizoctonia solani* AG1-IA is a devastating phytopathogen causing Rhizoctonia foliar blight (RFB) of soybean worldwide with yield losses reaching 60%. Plant defense mechanisms are complex and information from different metabolic pathways is required to thoroughly understand plant defense regulation and function. Combining information from different “omics” levels such as transcriptomics, metabolomics, and proteomics is required to gain insights into plant metabolism and its regulation. As such, we studied fluctuations in soybean metabolism in response to *R. solani* infection at early and late disease stages using an integrated transcriptomics-metabolomics approach, focusing on the regulation of soybean primary metabolism and oxidative stress tolerance.

**Results:**

Transcriptomics (RNAseq) and metabolomics (^1^H NMR) data were analyzed individually and by integration using bidirectional orthogonal projections to latent structures (O2PLS) to reveal possible links between the metabolome and transcriptome during early and late infection stages. O2PLS analysis detected 516 significant transcripts, double that reported in the univariate analysis, and more significant metabolites than detected in partial least squares discriminant analysis. Strong separation of treatments based on integration of the metabolomes and transcriptomes of the analyzed soybean leaves was revealed, similar trends as those seen in analyses done on individual datasets, validating the integration method being applied. Strong fluctuations of soybean primary metabolism occurred in glycolysis, the TCA cycle, photosynthesis and photosynthates in response to *R. solani* infection. Data were validated using quantitative real-time PCR on a set of specific markers as well as randomly selected genes. Significant increases in transcript and metabolite levels involved in redox reactions and ROS signaling, such as peroxidases, thiamine, tocopherol, proline, L-alanine and GABA were also recorded. Levels of ethanol increased 24 h post-infection in soybean leaves, and alcohol dehydrogenase (*ADH*) loss-of-function mutants of *Arabidopsis thaliana* had higher necrosis than wild type plants.

**Conclusions:**

As a proof-of-concept, this study offers novel insights into the biological correlations and identification of candidate genes and metabolites that can be used in soybean breeding for resistance to *R. solani* AG1-IA infection. Additionally, these findings imply that alcohol and its associated gene product *ADH* may have important roles in plant resistance to *R. solani* AG1-IA causing foliar blight.

**Electronic supplementary material:**

The online version of this article (doi:10.1186/s12870-017-1020-8) contains supplementary material, which is available to authorized users.

## Background

Rhizoctonia foliar blight (RFB) caused by *Rhizoctonia solani* anastomosis group (AG) 1 intraspecific group IA is a serious disease that causes rapid and severe destruction of soybean (*Glycine max* L. Merr) [1, 2]. Outbreaks of AG1-IA on soybean in Brazil, the southern states of the U.S.A. and China have caused yield losses of 30–60% [3, 4]. Protection against RFB is difficult as the use of less-susceptible cultivars is limited due to a lack of availability [1] resulting in the use of chemical pesticides. Because of the impact of RFB on agriculture, it is important to identify factors that regulate plant resistance; however, no studies have been published examining the molecular responses of soybean due to *R. solani* AG1-IA infection. Understanding the effect of *R. solani* AG1 on soybean defense pathways and their regulation will greatly assist breeding efforts towards the development of cultivars with improved resistance to RFB by applying biomarker-assisted selection.

There is growing interest in linking transcriptomics to metabolomics, which in turn could contribute to the comprehensive biological understanding that gene expression studies alone would otherwise not achieve. A large body of literature exists reporting on metabolic perturbations of plants caused by abiotic or biotic stresses through parallel or comparative analysis of microarray and metabolomics experiments [5–8]. More extensive analyses of the linkage between gene expression and metabolite biosynthesis is now possible given the recent advancements in tools and platforms to characterize various molecular entities of plant cells providing insights into the linkages between changes in gene expression and metabolite levels [8–12].

High-throughput multi-omics methods generate large and complex datasets that must be related to the biological system of interest, in this case soybean responses to RFB. Within this context, it is necessary to develop strategies that allow relevant biological processes to be described and easily interpreted in order to efficiently extract biologically significant information from analyses carried out with different omics profiling platforms. The bidirectional orthogonal projections to latent structures (O2PLS) is a new multivariate technique that integrates data from different datasets or “omics” levels (e.g., mRNA and metabolites), and supports multi-block bidirectional correlations [13, 14]. O2PLS is an extension of orthogonal projections to latent structures (OPLS) where as OPLS analyzes a single dataset, O2PLS assesses systemic trends across multiple datasets. This viable statistical method allows data to be integrated with equal weight allocated to each dataset irrespective of whether the number of data points in each dataset differs significantly. By observing the joint systematic variation, one can identify shared responses and utilize the model to predict biological responses between the datasets [9, 14]. Unlike pairwise correlation analyses that result in potentially hundreds of significant correlations [8, 15], O2PLS modelling reduces the number of correlations to those having the most dominant effects on the model [9]. Additionally, results can be interpreted based on both the predictive (joint variation) sources as well as on the unique, individual variables from each dataset [9]. To date, there are relatively few examples using O2PLS to integrate plant transcript and metabolite data [9, 11, 12] and, so far, no studies using O2PLS to uncover missing functional links between different “omics” levels for monitoring multi-level responses of plants to disease.

Phytopathogen infections lead to changes in secondary metabolism (i.e., metabolism not directly involved in maintaining growth, development or reproduction) as well as changes in primary metabolism (i.e., metabolism and its products required in all cells to maintain normal functions) that affect growth and development of the plant. While the regulation of defense responses has been intensively studied [16, 17], less is known about the effects of necrotrophic pathogen infection, such as *R. solani*, on primary metabolism. We have recently demonstrated the effectiveness of metabolomics for the analysis of plant primary metabolism during Rhizoctonia root rot and stem canker diseases of soybean hypocotyls and potato sprouts [18, 19] with results indicating that host plants reorganize their primary metabolism to mount defense mechanisms, while, pathogens might simultaneously manipulate plant metabolism to promote infection to support replication and spreading within the plant [20, 21]. The increased demands of redirection of primary metabolites involved in photosynthesis, carbohydrate and amino acid metaboism, glycolysis and TCA cycles suggest that reprogramming of vital functions in infected tissue occurs and is a necessary defense requirement [22–25]. Taken together, examining alterations in soybean primary metabolism to early and late infection stages of the foliar pathogen *R. solani* AG1 can provide insights on how to develop soybean lines with more resistance to foliar blight disease.

In the present study, we aimed to show that: 1) by integrating multiple omics levels, we were able to assess systemic and functional trends across transcripts and metabolites of soybean primary metabolism in response to a foliar necrotrophic fungal pathogen; 2) there is tight coordination in the context of known regulatory mechanisms and pathway level regulation of metabolism; and 3) the effect of a novel defense modulator can be examined using loss-of-function mutants.

## Results and discussion

**Fig. 1:**
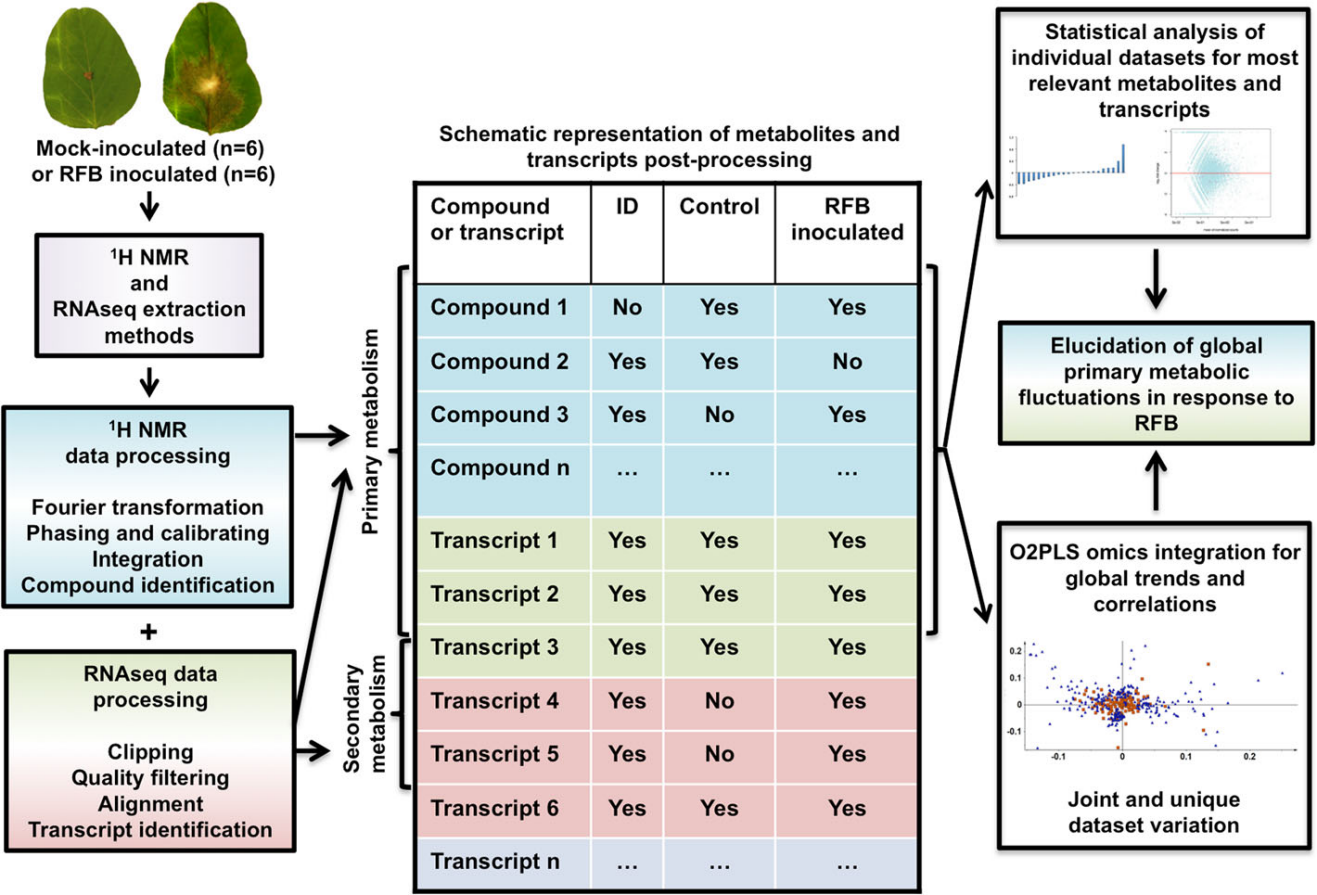
Overview of analytical approaches for integration of soybean metabolites and transcripts. Integrative approach applied to study soybean metabolite and transcript fluctuations in response to *R. solani* disease development. Data processing methods allowed for the identification of transcripts and metabolites as single and integrated datasets to find biomarkers within each dataset and trends between them for a global understanding of soybean responses to RFB

The complexity of plant metabolism necessitates the employment of multi-level approaches and integration of the obtained information for a comprehensive understanding of its regulation in response to stimuli. Initially, datasets were analyzed individually to identify substantially altered transcripts and metabolites of soybean responses to *R. solani*, followed by the discovery of joint variation between the two datasets by applying O2PLS, and focusing on the regulation of soybean primary metabolism (Fig. 1).

Previous studies examining soybean responses to fungal pathogens have mainly focused on fluctuations occurring in components of secondary metabolism, such as the phenylpropanoid, flavonoid and isoflavonoid pathways [17, 18, 26, 27]. Fluctuations in these pathways were observed during the soybean-*R. solani* interactions of this study (data not shown); however as few studies have examined the fluctuations in plant primary metabolism in response to *R. solani* [24], we focused on primary metabolism for this study. Additionally, to the best of our knowledge, no studies exist on soybean primary metabolism regulation in response to fungal attack using integrated omics approaches. Studies examining the primary metabolic responses of plants to fungal pathogens have suggested that rapid onset of certain genes or metabolites may be key components of plant resistance [21–23], emphasizing the role of primary metabolism in plant defense responses. The current study examined, for the first time, the overall metabolic responses of soybean to *R. solani* AG1-IA causing RFB disease.

### Overview of analyses

**Fig. 2:**
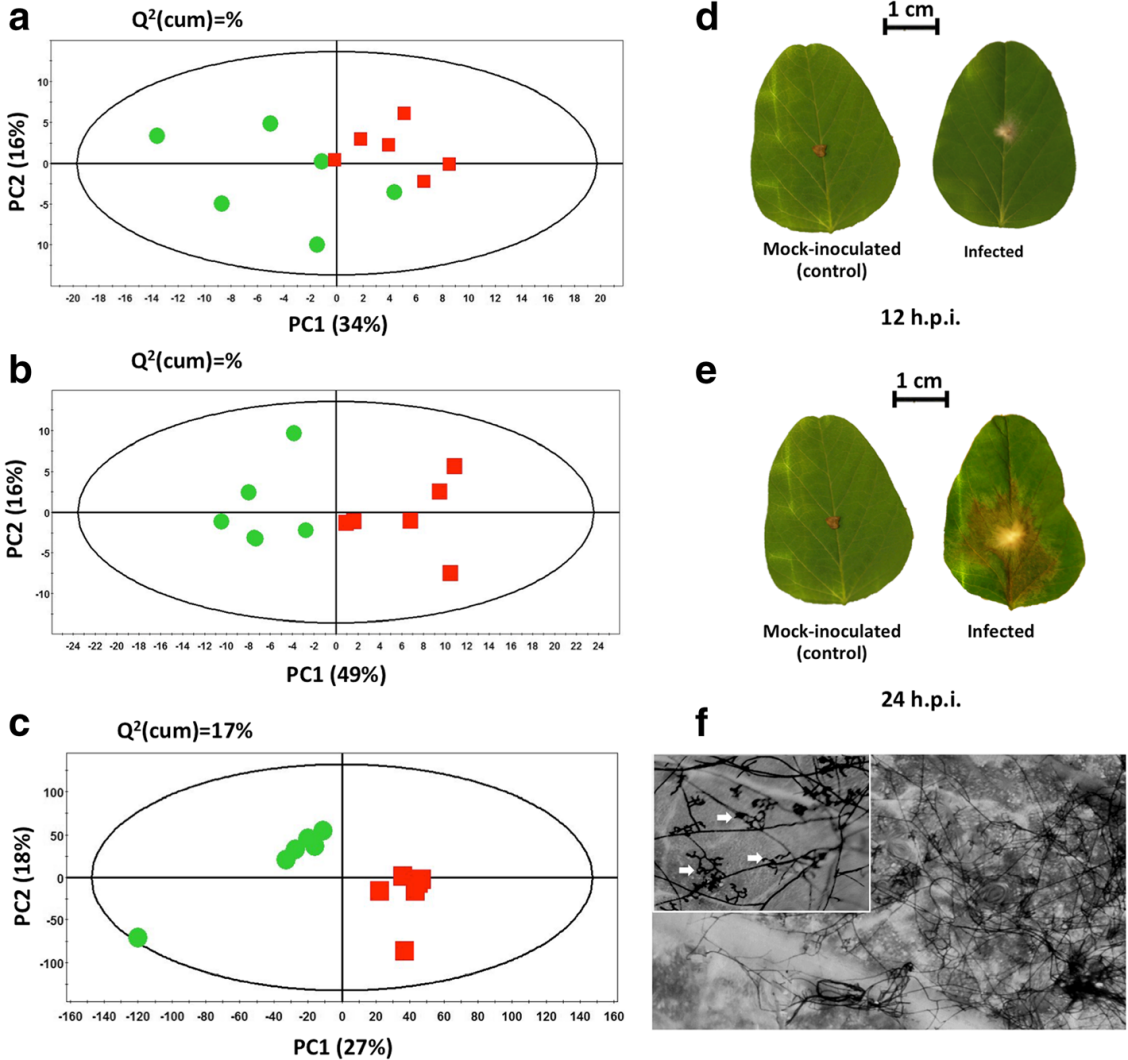
Soybean metabolite and transcript PCA score plots 12 and 24 h.p.i. with *R. solani* AG1-IA. PCA score plots of soybean metabolites from *R. solani* AG1-IA infected (*squares*) and control (*circles*) leaves 12 (**a**) and 24 (**b**) hours post-inoculation (h.p.i.), and RNAseq transcripts (**c**) 24 h.p.i. Ellipses represent Hotelling T^2^ with a 95% confidence interval. Six biological replicates were analyzed per treatment per time point. Soybean unifoliate leaves infected with *R. solani* AG1-1A and mock-inoculated controls 12 (**d**) or 24 (**e**) h.p.i. (**f**) Hyphal invasion and spread of *R. solani* over the soybean leaf and initials of infection cushions were visible at 12 h.p.i. (indicated by *arrows* in the *inset*)

Principal component analysis (PCA) was initially performed for the overview of ^1^H NMR and RNAseq datasets, the detection of outliers and trends, and the evaluation of robustness and reproducibility of the experimental protocol (Fig. 2). PCA revealed no outliers (*P* < 0.05) and a variable discrimination between the recorded metabolite profiles of mock-inoculated (control) and Rhizoctonia-infected leaves (Fig. 2). Moderate discrimination between the metabolic profiles of mock-inoculated (control) and infected leaves was observed 12 h post-inoculation (h.p.i.) (Fig. 2a) and is reflected by the early response to infection, signs of disease and development of several infection cushions on the leaf (Fig. 2d, f). A strong discrimination however, is observed 12 h later (24 h.p.i.) indicating the general disturbance of soybean metabolism **(**Fig. 2b) and establishment of the disease with full-blown symptoms (Fig. 2e). Based on these findings, the second time-point (24 h.p.i.) was selected for integration of NMR and RNAseq datasets. Similarly, PCA analysis of the RNAseq dataset (24 h.p.i.) revealed no outliers (*P* < 0.05) and a strong discrimination between the mock-inoculated and infected leaves (Fig. 2c), as supported by disease progression (Fig. 2e).

### Metabolite abundance changes in response to RFB

**Fig. 3:**
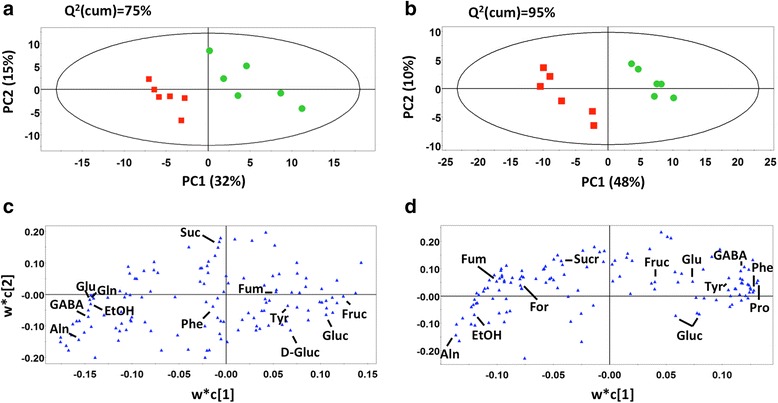
^1^H NMR metabolite partial least squares score and loadings plots 12 and 24 h.p.i. Partial least squares-discriminant analysis (PLS-DA) PC1/PC2 score (**a** and **b**) and loadings (**c** and **d**) plots of ^1^H NMR metabolite profiles 12 h.p.i. (**a** and **c**) and 24 h.p.i. (**b** and **d**) of *R. solani* AG1-IA infected (*squares*) and mock-inoculated control (*circles*) soybean leaves. Ellipses represent Hotelling T^2^ with 95% confidence intervals. Metabolites to the left increased during *R. solani* disease progression, while those on the right decreased in response to RFB compared to controls. Six biological replicates were analyzed per treatment per time point. Q^2^(cum), cumulative fraction of the total variation of the X’s that can be predicted by the components; R^2^X and R^2^Y, the fraction of the sum of squares of all X’s and Y’s explained by the components, respectively. *Aln*, alanine; *D-gluc*, D-glucose; *EtOH*, ethanol; *For*, formate; *Fum*, fumarate; *Fruc*, fructose; *GABA*, *γ*-aminobutyrate; *Gln*, glutamine; *Glu*, glutamate; *Gluc*, glucose; *Phe*, phenylalanine; *Pro*, proline; *Suc*, succinate; *Sucr*, sucrose; *Tyr*, tyrosine

**Fig. 4:**
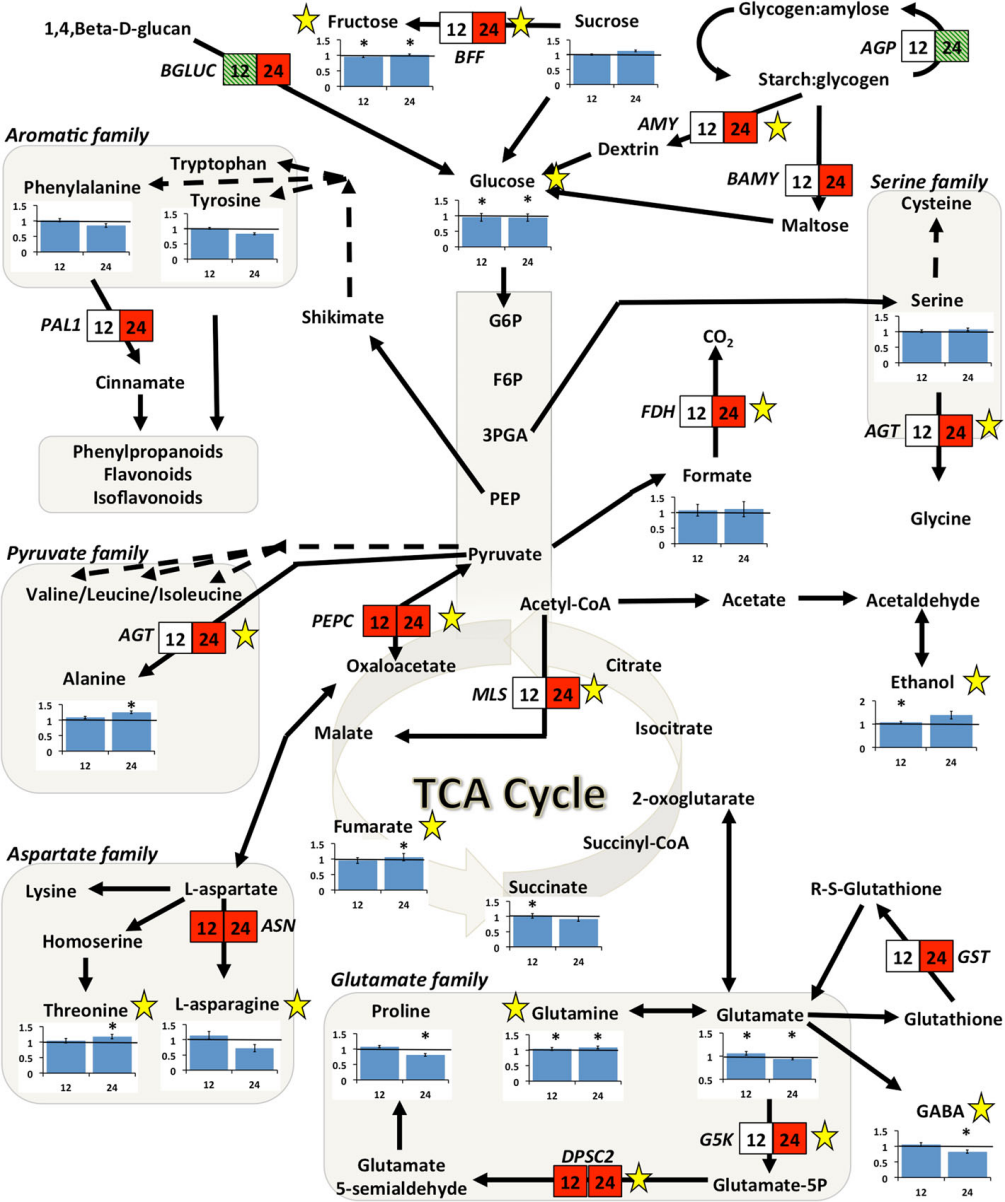
Metabolite and transcript pathway network analysis of soybean responses to *R. solani* AG1-IA. Gene-to-metabolite and metabolite-to-gene network fluctuations in soybean leaves in response to *R. solani* AG1-IA infection 12 and 24 h post-inoculation (h.p.i.). Metabolite fold changes are represented in bar graphs, and significant changes based on PLS-DA loading coefficients are indicated by an *asterisk* (*P <* 0.05). Transcripts are represented as *arrows* connecting metabolites. Transcript fold-changes (1.5 fold threshold; *P <* 0.05) based on qRT-PCR results are represented by *boxes* with 12 h.p.i. and 24 h.p.i. time-points represented on the left and right, respectively. Red illustrates an increase in transcript abundance in response to *R. solani* AG1-IA infection, while hatched green represents a decrease (*P* < 0.05 and fold change >1.5 or <−1.5). Capital lettering beside the boxes indicates gene name abbreviations (see Table 1). *Black arrows* without boxes represent transcripts detected in RNAseq, but not significantly affected by infection. *Solid arrows* represent single-step reactions, while *hashed arrows* represent multi-step reactions. *Stars* represent transcripts and metabolite bins that were identified as significantly altered using O2PLS analytical methods

Analysis of ^1^H NMR profiles of *R. solani* hyphae of similar surface area to that found at the necrotic lesions revealed a negligible impact of fungal metabolites on the total recorded metabolic profiles (Additional file 1: Figure S1). However, for accuracy, bins of fungal profiles were subtracted from the corresponding recorded bins of infected leaf profiles. Partial least squares-discriminant analysis (PLS-DA) has an improved ability for biomarker discovery compared to PCA (Fig. 3) [28]. Thus, here, biomarker discovery was based on PLS-DA (*P* < 0.05) to examine the metabolic fluctuations in soybean occurring during the early infection stage (12 h.p.i.) at which infection cushions develop (Fig. 2f), and during the late infection stage (24 h.p.i.) at which necrotic lesions develop. The tight clustering within the two treatments at both time points (Fig. 3a, b) is indicative of the robustness of the method and of the substantial differences between the metabolomes of control and infected leaves. A total of 126 bins representing seventeen uniquely identified metabolites of primary metabolism (Additional file 2: Table S1) were mapped onto metabolic pathways to view the temporal fluctuations in response to infection in a more dynamic manner (Fig. 4). Fluctuations of soybean metabolites within the glycolysis pathway, TCA cycle, starch and sucrose metabolism, and amino acid biosynthesis showed similar trends at early (12 h.p.i) and late (24 h.p.i.) stages of infection (Fig. 4; Additional file 2: Table S1) with the exception of GABA, L-asparagine, L-glutamate and proline, which increased and then decreased at 12 h.p.i. and 24 h.p.i. compared to controls, respectively.

### Effect of RFB on soybean transcript abundance

High-throughput sequencing allows for an in-depth analysis of the genome or transcriptome, however limits the number of biological replicates that can be sequenced in similar conditions (i.e., in one lane). Barcoding of samples permits pooling of multiple replicates allowing for the use of shallow-end sequencing to study a biological system. Due to the diminishment of the number of reads per sample, shallow-end sequencing can effectively be used to determine the fluctuations occurring in differentially expressed (DE) transcripts across a larger array of conditions than typically studied [29, 30]. It has been suggested that analyzing the top 2500 transcripts can in fact cover over 80% of the biological information in an Arabidopsis transcriptomic project [30], and as little as 100,000 reads per samples are required for accurate prediction of mRNA fluctuations in human studies [31] suggesting that shallow-end RNAseq can provide substantial information. In this study, shallow-end RNAseq was used to determine soybean responses to *R. solani* 24 h.p.i. and resulted in 3 M reads per treatment representing a total of 12,926 expressed genes (Additional file 3: Table S2), with expressed genes defined as the transcript having a minimum of two reads per sample and detected in a minimum of 4 out of 6 biological replicates in one treatment [32, 33]. Reads aligning to the *R. solani* genome represented less than 2% of the total reads and as such were not analyzed further. Low read counts is a common occurrence in dual plant-pathogen sequencing projects as typically the number of pathogen cells is much lower than those of the plant [32, 34].

**Fig. 5:**
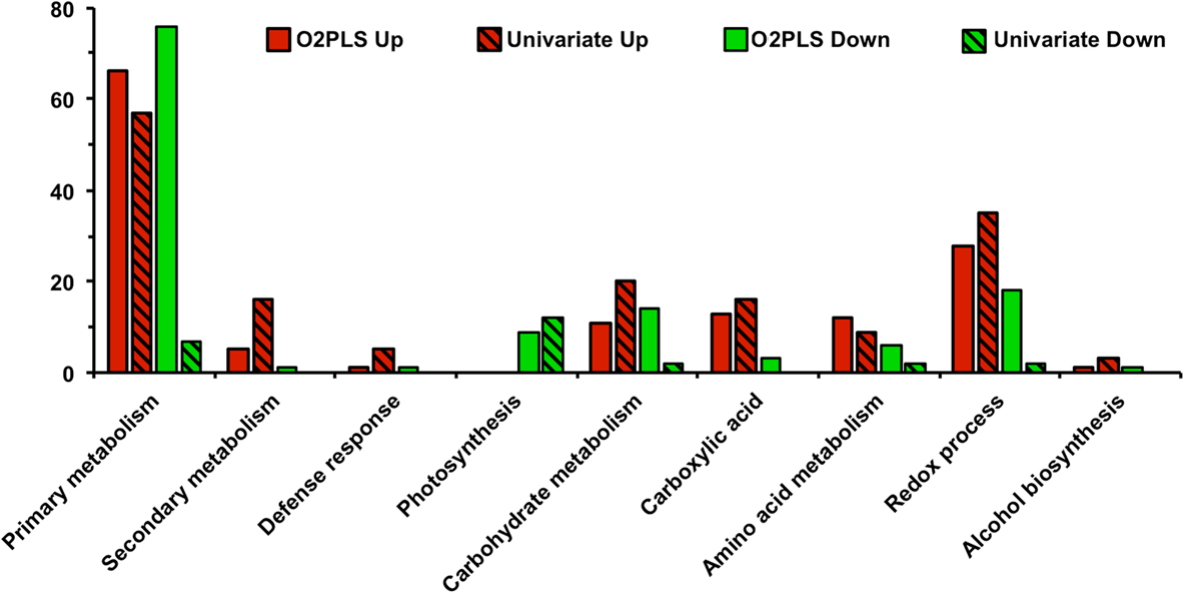
Fluctuations in gene ontology terms applying univariate analyses. Information on transcript data was obtained from the Panther database (http://www.pantherdb.org/)

A total of 258 DE genes were detected between infected and mock-inoculated leaves based on traditional univariate statistical analyses (Additional file 4: Table S3). These results are similar to RNAseq analyses of other plant-pathogen interactions [32]. Of the 258 DE genes, 79% were up-regulated in response to infection, out of which 16% could only be detected in infected plants, possibly due to detection limits. Enrichment tests of Gene Ontology (GO) terms [35] among the DE genes revealed the functional differences of the DE genes upon *R. solani* AG1-IA infection: a decrease in transcription of soybean genes functioning in photosynthesis was observed, whereas an increase in genes involved in secondary metabolism, redox reactions and carbohydrate metabolism was seen 24 h.p.i. (Fig. 5; Additional file 4: Table S3).

Quantitative real time-PCR (qRT-PCR) validated RNAseq results for genes involved in primary metabolism (Table 1) and showed similar expression patterns of the transcripts compared to RNAseq. A general trend of up-regulation of transcripts was observed (Fig. 4) implying an increased need for their products or downstream products. An exception was beta-glucosidase, whose abundance fluctuated (Table 1; Fig. 4). Validation of randomly selected transcripts also confirmed the results of RNAseq data (Additional file 5: Table S4).

**Table 1:** Fold change values of soybean genes affected by *R. solani* AG1-IA infection and involved in primary metabolism

Pathway	Gene ID	Gene annotation^a^	RNAseq Fold Change^b^	qRT-PCR Fold Change^c^ (*P* value)	
			(*P* value)	12 h	24 h
Starch metabolism	GLYMA08G45210	Alpha-glucan phosphorylase (*AGP*)	0.305 (0.0196)	0.793 (0.3489)	0.574 (0.0216)
	GLYMA04G01950	Alpha-amylase (*AMY*)	5.898 (0.0052)	1.059 (0.6037)	2.190 (<0.0001)
	GLYMA15G10480	Beta-amylase (*BAMY*)	2.800 (0.0200)	1.523 (0.0854)	1.509 (0.0249)
Carbohydrate metabolism	GLYMA05G04290	Beta-fructofuranosidase (*BFF*) or invertase	39.717 (<0.0001)	0.909 (0.3517)	1.763 (0.0196)
	GLYMA12G05780	Beta-glucosidase (*BGLUC*)	INF^d^ (0.0319)	0.317 (0.0164)	INF (<0.0001)
TCA cycle	GLYMA19G01200	Formate dehydrogenase (*FDH*)	7.092 (0.0020)	1.080 (0.6075)	1.453 (0.0212)
	GLYMA17G13730	Malate synthase (*MLS*)	10.718 (<0.0001)	1.249 (0.5463)	2.145 (0.0094)
	GLYMA01G23790	Phosphenolpyruvate carboxykinase 1 (*PEPC*)	INF (0.0058)	INF (0.0487)	INF (<0.0001)
Amino acid metabolism	GLYMA03G04990	Alanine-glyoxylate transaminase (*AGT*)	10.792 (0.0201)	1.164 (0.7345)	2.592 (0.0003)
	GLYMA02G39320	Asparagine synthetase (*ASN*)	INF (0.0038)	2.178 (0.0403)	2.159 (0.0277)
	GLYMA01G24530	Delta 1-pyrroline-5-carboxylate synthase 2 (*DPSC2*)	INF (<0.0001)	4.450 (0.0424)	3.486 (0.0003)
	GLYMA03G12240	Glutamate-5-kinase (*G5K*)	25.089 (0.0084)	1.289 (0.9217)	3.806 (0.0001)
	GLYMA19G36620	Phenylalanine ammonia lyase 1 (*PAL1*)	6.144 (0.0095)	1.362 (0.1645)	2.588 (<0.0001)
Glutathione metabolism	GLYMA10G33650	Glutathione-S-transferase (*GST*)	INF (0.0200)	1.578 (0.3678)	8.812 (<0.0001)

^a^Gene annotations based on the SoyBase database

^b^RNAseq fold change values based on pairwaise comparisons using the negative binomial test and an FDR correction <0.1 using Benjamini-Hochberg multiple corrections [87]

^c^qRT-PCR fold changes based on [81] efficiencies and *P* values based on pairwise comparisons using Student’s *t* test comparisons

^d^INF represents transcripts that were detected in infected samples, and not detected (below the detection threshold) in control samples

Transcript changes were deemed statistically and biologically significant if *P* < 0.1 for RNAseq or *P* < 0.05 and fold changes were >1.5 or <−1.5

### Metabolomics and transcriptomics integration

**Fig. 6:**
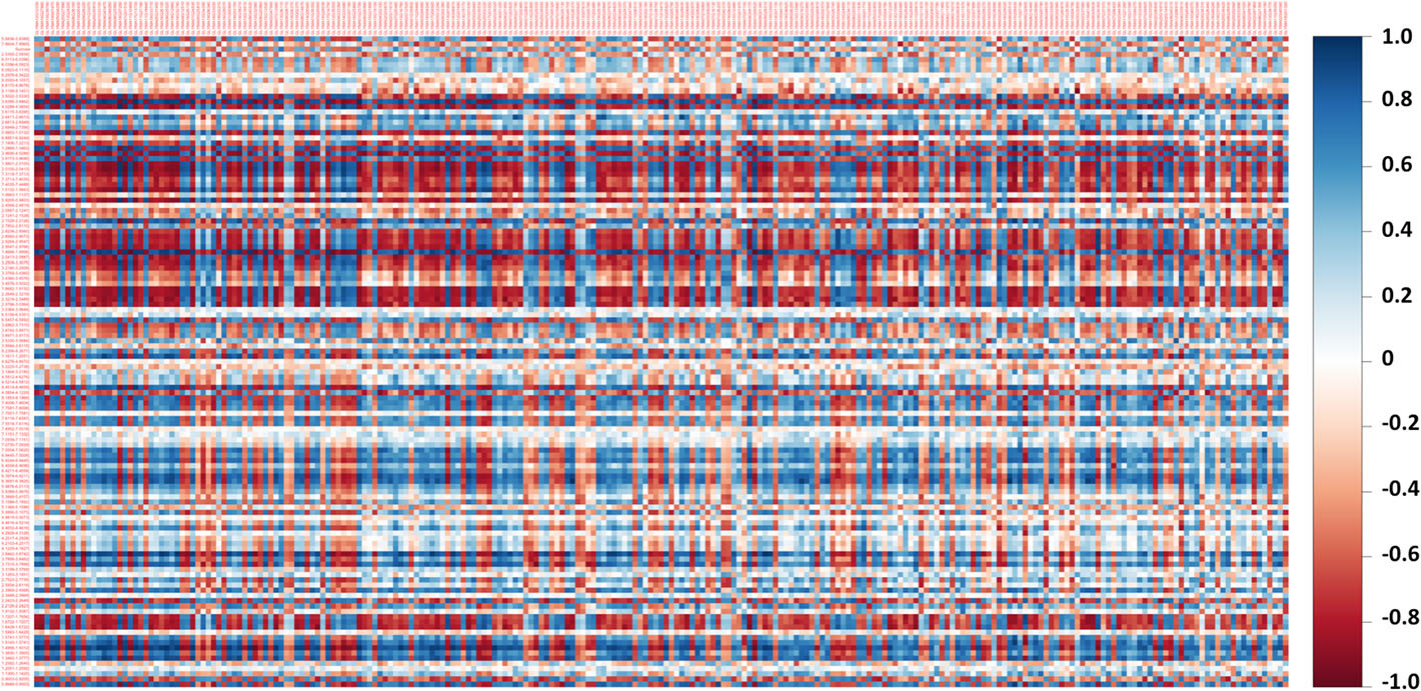
Heatmap of Pearson pairwise correlations between metabolites and transcripts. Correlations were calculated between the 126 metabolite bins and the top 241 transcripts based on variable importance (VIP) values using O2PLS analysis. *Blue* indicates a strong positive correlation between metabolites and transcripts, while *red* indicates a strong negative correlation. Refer to Additional file 6: Table S5 for metabolite and gene names

**Fig. 7:**
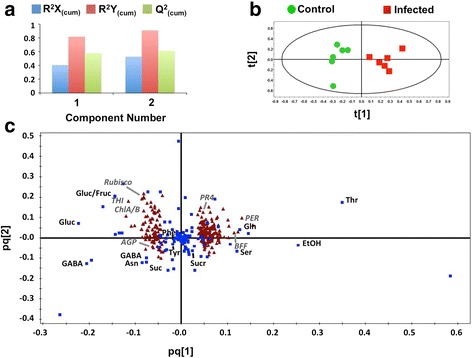
O2PLS integration of soybean metabolites and transcripts in response to *R. solani* AG1-IA. O2PLS integration performance overview plot (**a**), joint score plot (**b**) and loadings plot (**c**) of metabolites and transcripts involved in primary metabolism. All 12,926 transcripts were used for model development and validation (Q^2^). The top 241 transcripts from primary metabolism based on O2PLS variable importance >2 were chosen for correlations between ^1^H NMR and RNAseq integration for better visualization of trends between the two datasets (**c**). **a** Overview of cumulative predictive and orthogonal variation explained by the first two variables for the transcripts (R^2^X_(cum)_) and metabolites (R^2^Y_(cum)_), and the predictive power of the model (Q^2^_(cum)_). **b** Joint score plot of the transcript scores (t). **c** Joint loadings plot from the transcript (p) and metabolite (q) loadings blocks of the top 241 transcripts and all metabolites. Transcripts (*triangles*) and metabolites (*squares*) represent individual transcript and metabolite loading values (Additional file 8: Table S7 and Additional file 9: Table S9). Variables located to the left of the y-axis represent transcripts and metabolites that decreased, while those on the right increased, compared to controls in response to *R. solani* disease progression. Metabolite abbreviations: *Asn*, asparagine; *EtOH*, ethanol; *Fruc*, fructose; *GABA*, *γ*-aminobutyrate; *Gln*, glutamine; *Gluc*, glucose; *Phe*, phenylalanine; *Ser*, serine; *Suc*, succinate; *Sucr*, sucrose; *Thr*, threonine; *Tyr*, tyrosine. Transcript abbreviations: *AGP,* alpha-glucanphosphorylase; *BFF,* beta-fructofuranosidase; *ChlA/B,* chlorophyll A/B binding protein; *PER,* peroxidase; *PR4,* pathogenesis-related protein 4; *THI,* thiamine biosynthesis

Aiming to discover signatory genes and metabolites involved in soybean defense against RFB and to highlight functional links between them, data integration using pairwise correlations and O2PLS was performed. Pairwise correlation analysis revealed 382 very strong correlations (*r* ≥ 0.9 or ≤ − 0.9) and an additional 2494 strong correlations (*r* ≥ 0.8 or ≤ − 0.8) between metabolites and transcripts (Fig. 6; Additional file 6: Table S5). This large number of correlations results in challenging interpretation of which are the most biologically significant. As such, O2PLS analysis was performed on both datasets to reduce the noise and number of dominant correlations allowing for more confident biological interpretation and predictions. O2PLS identified 2 latent variables in the predictive dataset, with the transcript-predictive structures accounting for 52.9% of the total variation in the transcript dataset, the metabolite-predictive structures accounting for 90.9% of the total variation in the metabolite dataset, and a cumulated predictive power (Q^2^_(cum)_) of 61.3% (Fig. 7a). O2PLS score plots of the combined transcriptomics-metabolomics dataset revealed a tight clustering of the two treatments (Fig. 7b), results similar to those observed when analyzing the datasets alone. With the significance threshold set at a confidence level of 99% or 90% for transcripts and metabolites, respectively, the identified loading coefficients thresholds were lower for the transcriptomics dataset than for the metabolomics dataset (Additional file 7: Table S6). Based on loading coefficients, O2PLS analysis detected a total of 516 significant transcripts in the first two latent variables (Additional file 8: Table S7), double the transcript number that was found in the univariate analysis, while a total of 20 substantially altered metabolite bins were observed (Additional file 9: Table S8).

As a proof-of-concept, and in order to better visualize the functional links (i.e. correlations) between the metabolome and transcriptome, a reduced dataset was created composed of transcripts and metabolites involved in soybean primary metabolism, similar to methods done elsewhere [12]. The selection of transcripts was based on their involvement in primary metabolism using GO terms and O2PLS variable importance (VIP) values above 2. In total, 241 transcripts were selected (Additional file 10: Table S9), whereas all 126 bins of ^1^H NMR spectra were used. The reduced subset was then subjected to O2PLS to improve the visualization of their correlations that were generated in the fully integrated analysis (i.e. 12,926 transcripts by 126 metabolite bins) using variable loadings (Fig. 7c; Additional file 6: Table S5). Strong correlations could be seen between glucose and transcripts involved in photosynthesis (*Rubisco, PSII*), while negative correlations were observed between glucose and transcripts for thiamine biosynthesis (*THI*) (Fig. 7c). Of particular interest, were the correlations of alpha-glucan phosphorylase (*AGP*), a transcript involved in starch formation, with GABA, phenylalanine and tyrosine, all known for their roles in stress responses [16, 18, 21]. Moderately strong correlations (*r* 0.78 and 0.81) between peroxidase (*PER,* GLYMA16G27990) and beta-fructofuranosidase (*BFF,* GLYMA05G04290) with ethanol (Fig. 7c; Additional file 6: Table S5) were detected. Their importance may have been over-looked as their pairwise correlation values were below <0.9 and would not have been deemed significant (Fig. 7; Additional file 6: Table S5). The exact biological interpretation of these correlations remains unclear, and whether these metabolites/transcripts can in fact be used as biomarkers for each other requires further validation under various growth and biological conditions.

### Univariate vs. multivariate comparisons

**Fig. 8:**
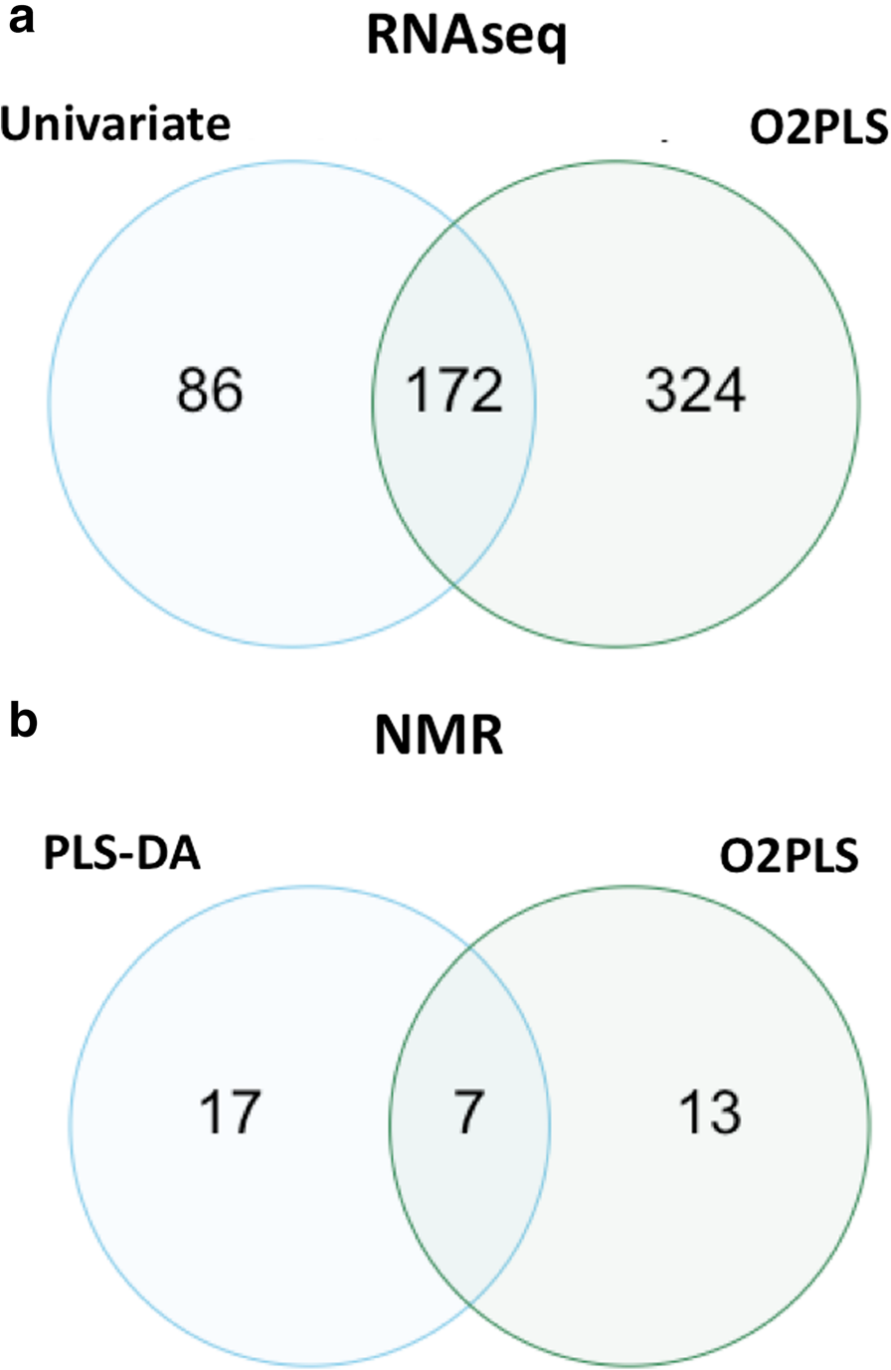
Venn diagrams of altered transcripts and metabolites. Overlap of differentially altered transcripts (**a**) and metabolites (**b**) between traditional and O2PLS analyses

Differences were observed between analyses of the datasets as stand-alone versus integrated with some transcripts or metabolites being identified as significant in one analytical method or both (Fig. 8), results similar to those reported elsewhere [9]. This can be observed in Fig. 4 with some differentially expressed transcripts and metabolites that are coordinately modulated in response to RFB using only traditional analyses, or using O2PLS analysis (indicated by yellow stars). Differences between univariate and multivariate analyses are not unexpected due to the methods by which the analyses calculate variation within the data: univariate analyses will calculate the variation between the two treatments, whereas O2PLS methods will calculate the direction within the two datasets (X and Y, or in this case transcripts and metabolites, respectively) that has the largest amount of variability [9]. Despite the differences, similar trends were observed between the two methods (data not shown).

### Biological interpretation

#### Metabolite fluctuations

Different temporal shifts occurred in the majority of metabolites and DE transcripts that were tightly associated suggesting that the metabolites are being utilized faster than they can be synthesized, or that their biosynthetic pathways are being shunted towards other products (Fig. 4). Exceptions were formate and ethanol whose associated transcripts were expressed, but not differentially in response to *R. solani* infection. Formate is a known fungitoxin [36], and a trending increase in its abundance at both time points may indicate an important role at later stages of infection. Ethanol levels, but not transcripts encoding alcohol dehydrogenase (*ADH*), increased before the appearance of necrotic lesions (12 h.p.i.) suggesting that ethanol may be a reliable disease biomarker.

**Fig. 9:**
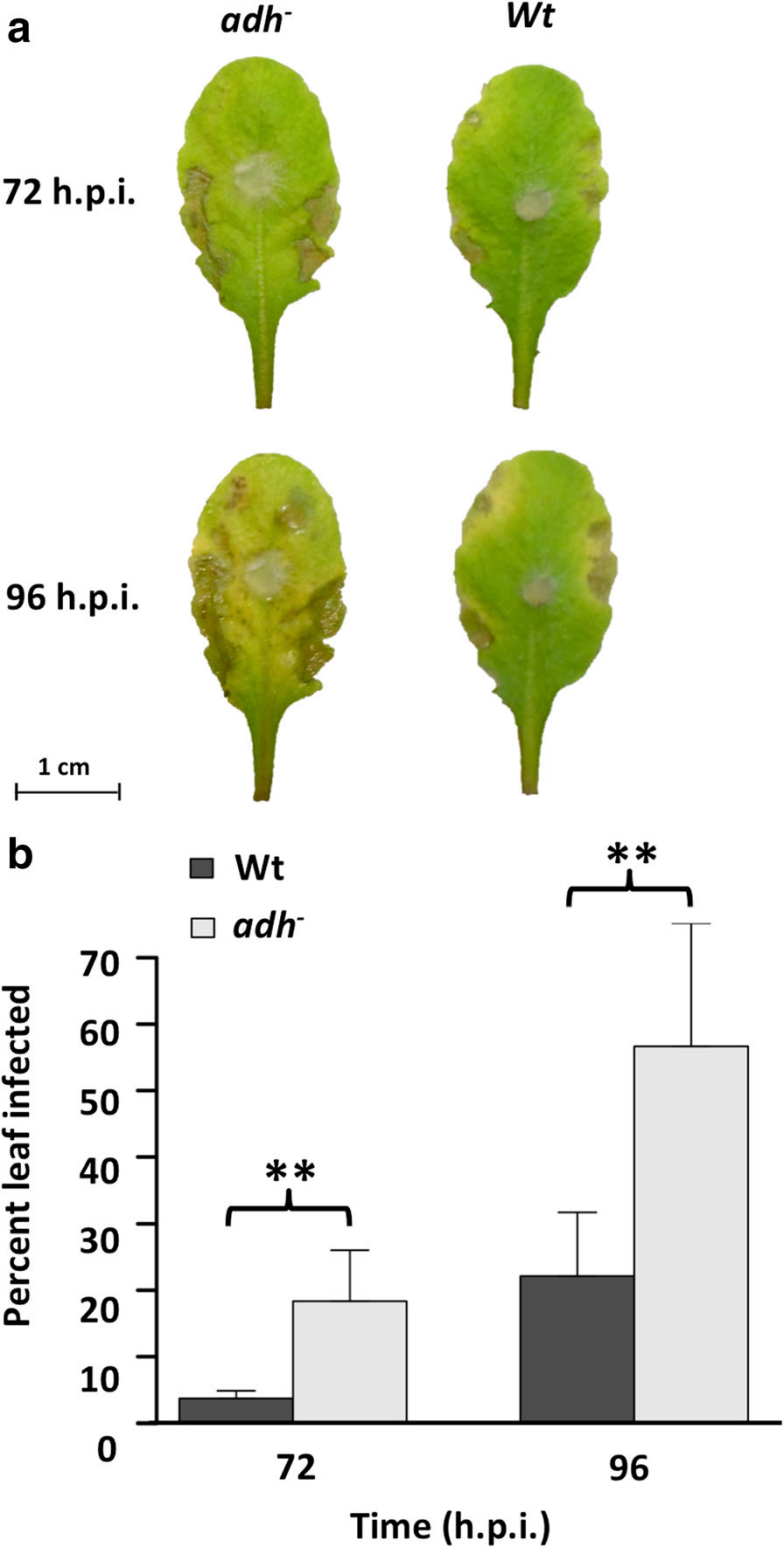
Role of alcohol dehydrogenase loss-of-function on *R. solani* AG1-IA infection in Arabidopsis. Generalized symptoms of RFB (**a**) and average percent infected area of leaves (**b**) in Arabidopsis wild type (Wt) and mutant (*adh* ^*−*^) plants 72 and 96 h post-inoculation. *Asterisks* denote significant differences at *P <* 0.05 using Student’s *t* test comparisons

To estimate the degree by which transcript changes and disease development occur, we selected Arabidopsis plants with an ADH loss-of-function. An increase in overall necrosis was observed in the mutants compared to controls (Fig. 9) suggesting that alcohol dehydrogenase and ethanol may have a role in RFB resistance. Consistent with our results, woody and herbaceous plants exposed to abiotic stress accumulated substantial levels of ethanol under aerobic conditions [37]. On the other hand, transcripts encoding soybean *ADH* were present in the RNAseq data, but were not affected during *R. solani* AG1-IA infection (Fig. 4; Additional file 3: Table S2) despite increases in the amount of ethanol produced during infection, suggesting that post-transcriptional or -translational modifications may play a role in ethanol production in soybean.

One may argue that ethanol presence in infected soybean leaves signals an anaerobic state or is fungal-derived. In our study, ethanol was also present in mock-inoculated leaf samples (controls) where ample oxygen was available to the leaves. Additionally, the levels of pathogen-derived metabolites were below the detection threshold or minimal when analyzing similar amounts of *R. solani* hyphae to those present on infected soybean leaves using ^1^H NMR (Additional file 1: Figure S1). Although the exact origin and mechanism(s) by which ethanol and alcohol dehydrogenase were up-regulated are not fully understood, this study is the first to demonstrate that they may have a role in plant defense against necrotrophic pathogens. While ADH activity has been reported to modulate the susceptibility of barely to the biotrophic obligate parasite *Blumeria graminis* and may support biotrophy by maintaining glycolytic metabolism in pathogen-stressed barley [38], this may not be the case with necrotrophic fungal pathogens such as *R. solani*. Supporting evidence shows that plants have differential mechanisms to respond to different necrotrophic and biotrophic pathogens [39, 40]. Taken together, our study suggests that ethanol may have an important role in plant resistance to the necrotrophic fungal pathogen *R. solani* and may help modulate plant resistance to RFB.

#### Transcript fluctuations

Although a portion of the transcripts can be linked directly with metabolites in primary metabolic pathways, this was not the case for all transcripts. These include some pathogen-responsive genes involved in defense and stress responses (Additional file 4: Table S3), such as glutathione-S-transferase and glutathione-peroxidase, two well-established genes with antioxidant capacity [16]. Interestingly, no up-regulation of ascorbate-related genes was observed, suggesting that glutathione was acting independently of the glutathione-ascorbate cycle [41] or that increases in ascorbate can occur in the absence of an increase in its transcript abundance [42, 43]. Several soybean peroxidases were up-regulated in response to RFB. This was expected as plants exposed to stress up-regulate their overall peroxidase activity resulting in plant defense either passively by building up stronger cell walls or actively via production of ROS molecules during the oxidative burst [16, 21].

Many compounds have ROS quenching capabilities in plants, such as thiamine, tocopherol and tocotrienol. Thiamine was implicated in Arabidopsis resistance to *Sclerotinia sclerotiorum,* possibly by limiting the effects of oxalate suppression on ROS signaling [44]. Thiamine transcripts were up-regulated in response to *R. solani* infection (Additional file 4: Table S3), and whether this is a result of *R. solani* oxalate or is a response to the oxidative stress caused by necrosis is not clear and merits further investigation to determine its role in soybean defense and responses to necrotrophic pathogens. Transcripts leading to the biosynthesis of tocopherol and tocotrienol (vitamin E vitamers) were up-regulated in response to infection (Additional file 4: Table S3). Tocopherols have long been speculated to have an essential function in protecting photosynthetic organisms against photo-oxidative stress [45]. Increased transcript abundance of genes involved in tocopherol and tocotrienol biosynthesis in response to *R. solani* infection may reflect lipid peroxidation, a common occurrence during plant-pathogen interactions [46].

#### Fluctuations in photosynthesis, glycolysis and the TCA cycle in response to RFB

Pathogen infection often leads to the development of chlorotic and necrotic areas, and decreases in photosynthesis transcript abundance and photosynthetic assimilate production [47, 48]. Down-regulation of transcripts involved in photosynthesis at 24 h.p.i. (Additional file 4: Table S3) was observed, implying a decrease in photosynthetic activities. This was paralleled by increases in transcript abundance of genes involved in starch and carbohydrate catabolism, as well as decreased levels of sucrose cleavage products (i.e. glucose and fructose) as early as 12 h.p.i. (Table 1; Fig. 4). This suggests either an increase in soybean energy demands, a typical plant stress response to disease [21], or manipulation of the plant sugars by the pathogen to promote infection.

A common trend is observed for the rapid increase in levels of invertases (e.g., beta-fructofuranosidase) after plant infection by bacteria and fungi (Table 1 our study; [49]). Similar to other necrotrophic pathogens, *R. solani* has faster growth on sucrose or other disaccharides as the carbohydrate source than when grown on their cleavage products [50]. Therefore, from the plant’s point of view, it is advantageous to modulate sugar partitioning and limit sucrose availability, thus limiting *R. solani* growth. Moreover, during infection invertase activity triggers plant defense responses such as induction of defense-related gene expression, callose deposition and reduction of photosynthesis or cell death [49, 51], and increases in invertase transcript abundance are often correlated with the role of hexoses as signaling molecules for defense gene activation [49]. Beta-glucosidase, which degrades beta-glucan to glucose, and whose transcript abundance increased at the late infection stage, may have had an important role in modulating soybean glucose concentrations; however, we cannot rule out the possibility that it also may have had a role in the degradation of *R. solani* hyphae, as beta-glucan is a common component of hyphal cell walls [52]. How carbohydrates and their presence as mono- or di-saccharides influence soybean resistance to RFB and other pathogens, and how they affect microbial growth, requires further investigation.

During stress responses as a result of pathogen invasion, plant defense mechanisms display coordinated fluctuations of genes and metabolites involved in glycolysis and the TCA cycle in an attempt to adapt to stress [24, 25, 53]. Despite the increases in storage carbohydrate catabolic genes, the majority of genes involved in glycolysis and the TCA cycle were not differentially expressed in response to RFB (Fig. 4). Several factors might help to explain this: 1) the timing of sampling (24 h.p.i.) was not the optimal time point to detect transcriptional changes; 2) other biological factors such as post-transcriptional or post-translational modifications occurred [42, 43]; 3) sufficient energy sinks were available to combat the infection at this time point; or 4) the genes require more time for differential expression to be detected. Homeostasis of metabolic pathways, particularly primary metabolic pathways, is essential for survival and the timing of induction or suppression of these pathways is most likely crucial for survival [21].

#### Fluctuations in amino acid biosynthesis in response to RFB

In response to infection, the strong demand to obtain carbon will likely shuttle amino acids into energy generating pathways such as the TCA cycle [21]. It has been proposed that plants may mobilize some nitrogen sources away from infection sites to deprive pathogens of nutrients. This diversion leads to drastic changes in source-sink relationships [54]. Shifts in amino acid concentrations with some being up-regulated and others down-regulated during early and late plant responses to *R. solani* infection are similar to what has been reported in other pathosystems [55, 56]. In this study, amino acid fluctuations (Fig. 4) could be grouped into: 1) those that were up-regulated (glutamine, alanine, threonine and serine) at both time points; 2) those that were down-regulated (phenylalanine and tyrosine) at 24 h.p.i.; and 3) those that were up-regulated as early as 12 h.p.i. and then decreased at 24 h.p.i. (L-asparagine, glutamate, proline and GABA). Fungi derive amino acids from plants by recycling or via proteolysis [57], and the different temporal fluctuations of amino acids observed in this study may indicate differential amino acid requirements for *R. solani* due to an inability to synthesize certain amino acids, different amino acid requirements during different infection stages (onset versus necrosis), or differential amino acid requirements of the plant during defense responses.

In many higher plants, the nitrogen-rich amino acids asparagine and glutamine represent central intermediates in nitrogen as they contribute to nitrogen transport, and their encoding genes are up-regulated under biotic stresses [22, 23]. Asparagine along with proline, GABA and its precursor glutamate, and the non-polar amino acids phenylalanine and tyrosine, exhibited slight increases at the onset of fungal infection and decreases at the late infection stage, although levels of transcripts associated with their production (except for GABA and tyrosine) increased in abundance at the late infection stage (Table 1, Fig. 4). This may suggest that early on, the infection sites represent strong local metabolic sinks that drain nutrients from uninfected healthy regions, and as the infection becomes established different scenarios are favored: they are utilized downstream and the biosynthesis rate is slower than the consumption rate [21], they are being utilized by *R. solani,* or *R. solani* is capable of manipulating its host’s metabolism for particular amino acid homeostasis [57, 58].

Proline and GABA have been shown to be important amino acid signaling molecules during plant stress [21, 59]. GABA is known to help support energy requirements via the GABA shunt pathway during times of high-energy requirements [60] and high oxidative stress [61, 62]. The decrease in both GABA and its precursor glutamate at the late infection stage coupled with the increase in antioxidant producing genes (RNAseq only) discussed earlier indicate a state of high oxidative stress that could be overcome by shifting these resources through the GABA shunt pathway.

Similarly, proline is a stress-related metabolite that has been shown to have a role in stabilizing and protecting proteins and membranes from cellular ROS during fungal infection [63]. The oxidation of proline provides electrons for mitochondrial respiration resulting in increased energy supplies for the TCA cycle [63]. The decrease in proline observed during the necrotic stage of infection may suggest an increase in energy requirements at this stage causing proline to be shunted towards the TCA cycle, or an increased demand for ROS signaling, a common response during plant-pathogen interactions [16, 21]. Glutamine and alanine concentrations increased during *R. solani* AG1-IA infection. Similar findings were reported in rice infected with compatible but not incompatible *Magnoportha grisea* strains [23]. Alanine has also been linked to induction of programmed cell death in Concord grape (*Vitis labrusca)* cell cultures [64]. It is possible that successful penetration of the pathogen triggers an increase in alanine levels to promote cell death of the infected tissue, which *R. solani* then exploits to facilitate invasion.

Regulation of primary metabolic pathways is crucial for soybean to maintain vital functions in addition to regulating soybean defense responses to RFB and possibly altering oxidative stress responses. The integrative analysis of transcript and metabolite profiling using O2PLS provided a powerful tool to better understand gene-to-metabolite networks over a time course of *R. solani* infection of soybean by identifying: 1) significant transcripts and metabolites not deemed significant using traditional analytical approaches; 2) shared regulatory mechanisms through identification of joint systemic variation (example ethanol with beta-fructofuranosidase and peroxidase transcripts); and 3) reducing the number of significant correlations to those with the most significant effect on the biological system of interest (i.e. soybean-*R. solani*). Through validation experiments, we demonstrated changes within selected major biochemical pathways during disease establishment, and consequently, the identification of correlative biomarkers for genetic improvement and breeding assisted programs. We strongly believe that one important approach to gain knowledge in plant-microbe interactions is to combine results from different types of analyses, as done here.

## Conclusion

In conclusion, analyses of large multi-omics datasets represent one of the major challenges towards the understanding of the molecular regulation of a biological system [9, 12, 65]. As integrated omics studies are expected to expand our understanding on the molecular regulation of biological systems, including cause-and-effect and identification of correlative biomarkers [66], it will be important to compare different datasets using multiple analytical methods in order to avoid overlooking potentially important information.

## Methods

### Biological material and inoculation of soybean leaves

To study soybean responses to RFB disease, the fully sequenced model and reference cultivar Williams 82 (USDA-ARS, Washington, DC, USA) was used. Seeds were surface sterilized in 30% hydrogen peroxide for 7 min followed by three rinses in sterile water. Germinated seeds were planted in 5 cm pots containing AgroMix® G10 (Fafard Ltd.) and sand (1:1, *v*/*v*) with one seed per pot and placed in a growth cabinet with 12/12 h of day/night, 25/23 °C day/night temperatures, 210 photons μm^−2^ s^−1^, and humidity maintained at 65% throughout the entire day. Fully expanded unifoliate leaves (approximately 10 days post-planting) were detached and placed on dampened sterile filter paper in previously autoclaved Pyrex® dishes (25 × 15 cm).

To investigate the role of ethanol in RFB resistance, the wild type *Arabidopsis thaliana* (ecotype Bensheim, *Be-0*, TAIR germplasm #CS964) and the mutant (*ADH-R002,* TAIR germplasm #CS8102) with a truncated version of the alcohol dehydrogenase protein (described in Jacobs et al. [67] and Dolferus et al. [68]), were obtained from the Arabidopsis Biological Resource Center (ABRC, Ohio State University, Columbus, OH, U.S.A.). No over-expressing lines were available and thus only loss-of-function of ADH was examined. The seeds were surface sterilized in 2.75% sodium hypochlorite with 0.05% Tween® 20 for 20 min and rinsed 7 times with sterile distilled water. Three seeds of either wildtype or *adh* ^*−*^ mutants were pipetted into 10 cm pots containing Agro Mix® G10 (Fafard Ltd., St. Bonaventure, Canada) and grown under the same conditions as soybean. Pots were thinned to one plant per pot 2 weeks after planting.

A starter culture of a highly pathogenic *Rhizoctonia solani* AG1-IA strain ROS 2A4 (obtained from Dr. Paulo Ceresini, São Paulo State University, Brazil) was grown for 3 days on potato dextrose agar (PDA) from stock cultures kept in 20% glycerol at −80 °C. The inoculum for soybean consisted of *R. solani*-infested millet seeds prepared as follows: millet seeds obtained from Living World (Baie D’Urfé, Canada) were de-hulled, sterilized and placed as a single layer on the surface of one-week-old PDA fungal cultures originated from the starter cultures. Millet seeds became completely colonized by *R. solani* mycelia after one week of incubation at 24 °C. Sterile millet seeds placed on clean PDA plates for one week served as mock inoculum (control treatment). The inoculum for *A. thaliana* consisted of 3 mm plugs of 3-day-old *R. solani* strain ROS 2A4 grown on PDA. Sterile PDA plugs served as controls.

Soybean leaf inoculation was performed by placing one infested or sterile (control) millet seed in the middle of the detached unifoliate soybean leaf. One unifoliate leaf from each plant was used for infection treatments and the other for the mock-inoculation (control) treatments. The Pyrex dishes were wrapped in saran wrap and placed in a growth cabinet under the same conditions as plant growth described above.

*Arabidopsis thaliana* leaves from the second whorl of six-week-old plants were removed and inoculated with 3 mm plugs of 3-day-old *R. solani* ROS 2A4 grown on PDA or mock-inoculated with sterile PDA plugs (controls). PDA plugs were used for inoculation in Arabidopsis rather than colonized millet seeds to limit the effect of prior glucosinolate exposure (contained within the millet) on the Arabidopsis-*R. solani* interaction. Glucosinolates are important for Arabidopsis defense, and as such may have affected the interaction. Glucosinolates are not produced in soybean and as such would not have affected the soybean-*R. solani* interaction. Disease progression on Arabidopsis was monitored over a period of 96 h, and photos were taken every 24 h for necrosis analysis using ImageJ software version 1.49 [69] by thresholding and quantifying the amount of brown-yellow pixels and total leaf area [70]. Total necrosis and chlorosis were calculated as the percentage of the leaf (i.e., amount of brown-yellow pixels compared to total leaf area pixels) and results compared using Student’s *t* test comparisons with JMP software version 11.0 (SAS Statistics).

### Sampling

To study soybean metabolite and transcript fluctuations in response to *R. solani* infection, infected and mock-inoculated soybean leaves were harvested 12 and 24 h post-inoculation (h.p.i.). Samples were processed by cutting the leaf area containing the *R. solani* hyphae plus an additional 0.5 cm beyond the hyphae with a sterile scalpel, and frozen in liquid nitrogen. Similar size leaf areas from mock-inoculated leaves (control) were excised. Six excisions were pooled together for one biological replicate and a total of six replicates were analyzed per treatment. Transcripts and metabolites were extracted from the same samples to minimize variation between the metabolomic and transcriptomic datasets.

To control for the detection of *R. solani* metabolites in the infected leaves, hyphae of 12-h-old and 24-h-old *R. solani* cultures grown on PDA were harvested such that their amount was representative of the hyphal expansion area on the soybean leaves. Hyphae of six *R. solani* samples of similar size to those on the leaves were pooled together. Two biological replicates were done for the *R. solani* culture controls.

### Metabolite extraction and ^1^H NMR metabolomics analysis

Chemicals and reagents used for all experiments were of the highest grade commercially available. Deuterium oxide (D_2_O) for ^1^H NMR analyses was purchased from Sigma-Aldirch.

For metabolomics analysis, samples (12 and 24 h.p.i. and their respectively mock-inoculated control leaves) were ground in liquid nitrogen to a fine powder using a mortar and pestle. Polar metabolites were extracted from 200 mg of fresh tissue for leaves, or from the total harvest of the *R. solani* hyphae. Samples were freeze-dried for 24 h followed by addition of D_2_O (0.6 mL) in Eppendorf tubes (2 mL). Samples were sonicated for 30 min in a Branson® 5510 ultrasonic cleaner (Branson Ultrasonics) in the dark followed by a 3 h extraction at 250 rpm in the dark at room temperature. Finally, samples were centrifuged twice at 21,460 g for 1 h at 4 °C to remove debris and the supernatant placed in Eppendorf tubes (2 mL) and stored at −80 °C until NMR analysis. Two quality control (QC) samples were prepared for each treatment by combining equal volumes of sample extracts together.

Polar extracts of soybean leaves and *R. solani* hyphae were analyzed using ^1^H NMR as previously described [71] with some modifications. Briefly, NMR spectra were recorded using a Varian Inova 500 MHz NMR spectrometer (Varian) equipped with a ^1^H{^13^C,^15^N} triple resonance probe. A total of 128 transients of 64 K data points were acquired per sample, with a 2 s acquisition time and a 2 s recycle delay with pre-saturation of H_2_O during the recycle delay. Spectra were then Fourier transformed and the phase and baseline were automatically corrected using the Spectrus software v.12.01 (ACD/Labs). Binning is a commonly applied data-reduction method in spectroscopy, where spectra are divided in segments, the so-called bins, which are then integrated. Here, binning was performed using the intelligent bucketing option of the software, using a 0.04 ppm bin size and 50% width looseness. By performing intelligent bucketing, better clustering of spectra points can be achieved than performing conventional bucketing, thus strenghening the robustness of data. Identification of metabolites and peak annotation was based on shifts, coupling constant values, and comparisons with analytical standards that had been analyzed in the same system under identidical analytical conditions. The obtained combined NMR matrix of all samples was exported to MS Excel® and finally to SIMCA-P+ v.13.0.3.0 software (Umetrics) for statistical analyses.

### RNA extraction and data pre-processing

Total RNA from all treatments and time points (12 and 24 h.p.i. and their respective mock-inoculated controls) was extracted from 100 mg of infected or control soybean leaves using the RNeasy plant mini kit (Qiagen) following the manufacturer’s protocols. RNA quality was confirmed on a denaturing formaldehyde agarose gel (2%) and quantified using a NanoDrop ND-100 spectrophotometer (Thermo Fisher Scientific).

Total RNA (10 μg) from infected and control samples at the 24 h time point was used to isolate mRNA for RNA sequencing (RNAseq). RNAseq library preparation was performed following modified methods of Kumar et al. [72]. Briefly, mRNA was isolated using DynaBeads® (ThermoFisher Scientific), and first and second strand cDNA synthesis was done following the protocols of Kumar et al. [72] with AMPure® bead purification (Beckman Coulter). cDNA was fragmented using Fragmentase (NEB) with incubation at 37 °C for 20 min. End repair and A-tailing were performed using the NEB enzyme mix and Klenow 3′ to 5′ exo (ThermoFisher Scientific). Adapter ligation was done using 2X rapid T4 DNA ligase (NEB) and NEXTflex-96 RNA-seq barcode adapters 85 to 96 (BiooScientific). Samples were then PCR enriched using 1X Phusion High Fidelity PCR Master Mix (NEB), 0.67 μM each primer PE1/PE2 (Bioo Scientific) and 7.5 μL of the sample with the following thermocycle conditions found in Kumar et al. [72] for a total of 15 cycles. Individual library quality was assessed on a 2% agarose gel and quantified using a NanoDrop ND-1000 Spectrophotometer (Thermo Scientific). Equal amounts of each library were pooled together, quality and quantity confirmed using a bioanalyzer at the UC Davis Genome Center (University of California, Davis, CA, USA) and sent for paired-end sequencing on an Illumina HiSeq 2000 for 150 cycles at the UC Davis Genome Center (University of California, Davis, CA, USA).

Sequence data were filtered using the Illumina pipeline to separate reads belonging to different replicates by association with their respective Illumina barcodes. The quality of each library (sample) file was checked using FastQC version 0.10.1 [73]. Illumina adapters and barcodes were removed using the software Cutadapt version 1.2.1 [74], and further processed to obtain reads with cumulative quality scores above 20 and minimum sequence lengths of 40 bp using the Fastx-toolkit version 0.0.13 commands fastq_quality_filter and fastx_trimmer [75]. Sequences were aligned to the soybean genome and annotation v1.1 available on the JGI Genome Portal (http://genome.jgi-psf.org/) [76] and the *Rhizoctonia solani* AG1-IA genome Rhisol AG1-IA v1.0 available on NCBI (taxid: 983,506; BioProject Accession PRJNA51401) [77]. Sequences were aligned to the genome and annotated using the combined programs Bowtie2 version 2.1.0.0 [78] and TopHat2 v2.0.8b [79] with the following call: tophat2 --segment-length 18, −-segment-mismatches 3, −a 4, −m 1, −p 4, −-read-edit-dist 4, −g 1, −G [path to annotation file] [path to genome indices] [data file in fastq format]. Sequences aligning to the *R. solani* genome were removed prior to alignment to the soybean genome. To do so, a high amount of mismatches were allowed in the alignment due to the high genetic diversity found within *R. solani* anastomosis groups. This ensured the removal of any putative *R. solani* transcripts prior to alignment to the soybean genome. After mapping, sequences and isoforms were compared to the soybean reference transcriptome and counted using the HTSeq version 0.5.4p3 call: -m union, −s no, −t exon, −i gene_id [path to output sam file] [path to input sam file] [path to annotation file] [path to feature counts file] [80].

### RNAseq validation via qRT-PCR

Total RNA was extracted from soybean leaf samples (12 and 24 h.p.i. and their respective mock-inoculated controls) and processed in the same manner as described above. cDNA was synthesized with the iScript Advanced cDNA Synthesis kit for qRT-PCR (Bio-Rad Laboratories Ltd.) using 2 μg of total RNA from 12 h and 24 h time points of both infected and mock-inoculated soybean leaves. qRT-PCR was performed based upon genes that were differentially expressed (DE) at the 24 h time point RNAseq analyses. Briefly, 7 (Additional file 5: Table S4) genes were randomly chosen for validation of the RNAseq analyses and were analyzed at all time points (12 h, and 24 h post-inoculation) for all treatments. Another 14 DE genes of the primary metabolism pathways were selected to examine the effect of RFB on soybean primary metabolism, and also for visualization of metabolite fluctuations with the metabolomics data. qRT-PCR was done under the following conditions: each 20 μL reaction contained 1X SsoAdvanced Universal SYBR Green Supermix (Bio-Rad Laboratories Ltd.), 0.175–0.25 μM each primer (see Additional file 11: Table S10 for individual primer sequences and thermocycling conditions), and 600 ng cDNA. The thermocycling profile used as initial denaturation at 95 °C for 3 min, followed by 35 or 40 cycles of denaturation at 95 °C for 30 s, annealing for 30 s and extension at 72 °C for 40 s, followed by a dissociation curve analysis (see Additional file 11: Table S10 for individual primer sequences and thermocycling conditions). Gene expression was analyzed using the method of Zhao and Fernald [81] with normalization over the housekeeping gene UKN2 [82].

### Statistical analysis and biomarker discovery

#### Statistical analysis and biomarker discovery applying multivariate analysis for metabolomics

^1^H NMR data matrices were subjected to multivariate analyses using the SIMCA-P+ v.13.0.3.0 software for the detection of trends and biomarker discovery as previously described with minor modifications [83]. Briefly, PCA was initially performed for an overview of the data and the detection of outliers. The lower and upper α/2 percentiles were chosen as significance thresholds for the transcript and metabolite loading coefficients [9], with α = 0.90. The performance of the obtained models was assessed by the cumulative fraction of the total variation of the X’s that could be predicted by the extracted components [Q^2^(cum)] and the fraction of the sum of squares of all X’s (R^2^X) and Y’s (R^2^Y) explained by the current component. Fold changes in metabolite data were analyzed by comparing the total percent area under the curve of individual metabolites using Student’s *t* test comparisons using JMP software version 11.0 (SAS Statistics, Toronto, Canada).

#### Statistical analysis and biomarker discovery applying univariate analyses for qRT-PCR and transcriptomics

Normalization and pairwise differences in soybean RNASeq transcript counts were performed using the R version 3.0.2 [84] with package DESeq version 1.14.0 [85, 86]. Counts were normalized using the DESeq estimateSizeFactors function, and gene dispersion was determined using the function estimateDispersions. Pairwise comparisons for differences in normalized counts between treatments (6 biological replicates per treatment) were computed using the negative binomial test in the R package DESeq. The significance threshold was set at FDR <0.1 after correction for multiple comparisons using Benjamini-Hochberg correction [87], and a fold change of >1.5 or <−1.5 was used as a biological significance threshold. For identification of outliers within treatments, principle component analysis (PCA) was performed on the RNAseq using SIMCA-P+ v.13.0.3.0.

Data of qRT-PCR were analyzed using the efficiency calibrated mathematical model [88], where efficiency was calculated for each transcript using the method of Zhao and Fernald [81]. Differences in relative transcript abundance were determined using Student’s *t* test comparisons (*P* < 0.05) with JMP software version 11.0 (SAS Statistics), and biological significance with fold changes >1.5 or <−1.5.

#### Data integration-pairwise correlations and bidirectional orthogonal projections to latent structures (O2PLS)

Pearson pairwise correlations between metabolite bins and transcripts were calculated using Pearson’s correlations using R statistics version 3.0.2 [84]. Heatmap visualization of the pairwise correlations was done with the R package corrplot version 0.77 [89].

For the integration of transcriptomics with metabolomics dataset, a multivariate integrated omics model was built employing O2PLS, which has as main objectives the integration of multi-block datasets (i.e. X/Y) via the discovery of the jointed variation and the unique variation [9]. Initially, the metabolomics and transcriptomics datasets were merged in a single matrix in MS Excel, and then imported into SIMCA-P+ v.13.0.3.0. Transcriptomics data (12,926 transcripts) were assigned as X’s (block 1) and metabolomics data (126 bins) as Y’s (block 2). Since there are no studies on the integration between RNAseq and ^1^H NMR data, preliminary analyses showed that centering without scaling for both datasets gave the best results regarding the discriminatory and predictive ability of the obtained model and loading scattering (Additional file 12: Figure S2; Additional file 13: Figure S3 and Additional file 14: Table S11). In addition to optimization of scaling methods, the model was tested for over- and under-fitting leading to improper conclusions from the data. Therefore, to validate the model, Monte Carlo cross-validation was performed with 7 groups and 200 iterations [9, 90, 91]. Briefly, the method divides the data randomly into 7 groups and predictions are made from n-1 groups while the last group is put aside. Predictions are then compared to the true values of the excluded group and the prediction error sum of squares (PRESS) is determined. This is repeated until all groups have been excluded from the predictions once. This was done for various numbers of orthogonal components and the optimal number of components selected based on the most accurate/predictive scenario [9, 90, 91].

Variables of significant importance were selected based on variable loading coefficients, with significant thresholds set at α equal to 0.99 or 0.90 for transcripts and metabolites, respectively. The lower and upper α/2 percentiles were chosen as significance thresholds for the transcript and metabolite loading coefficients [9]. For clearer visualization of correlation trends in primary metabolism between the two datasets, the transcriptomics dataset was reduced to contain transcripts involved only in primary metabolism and to contain a similar amount of data points as the metabolomics dataset (126 metabolic bins). The reduced transcriptomics dataset contained transcripts involved in primary metabolism with variable importance values above 2 as determined by O2PLS integration (Additional file 10: Table S9), resulting in 241 transcripts and 126 metabolite bins for visualization of the correlations in primary metabolism. Reduction of datasets to pathways of interest when using integrative approaches allows for better observation of correlations between markers in the pathway(s) of interest [12]. Correlation trends between the two datasets in primary metabolic pathways were done by examining the reduced set O2PLS loadings plot (SIMCA-P+ v.13.0.3.0). The SoyBase database (soybase.org) was used for transcript GO term identification.

### Gene-to-metabolite and metabolite-to-gene network analysis

The effect of *R. solani* infection on soybean gene abundance (RNAseq and qRT-PCR) and metabolite fluxes was mapped onto the primary metabolic pathways by reconstruction of data available in the Kyoto Encyclopedia of Genes and Genomes (KEGG) database (http://www.genome.jp/kegg/) and previously published literature.

## Additional files

Additional file 1: Figure S1. Representative overlapping ^1^H NMR spectra of infected soybean leaves *and R. solani* AG1-IA controls. (PPTX 349 kb)

Additional file 2: Table S1. Soybean normalized metabolite bin values during RFB disease development at 12 h.p.i and 24 h.p.i. detected using ^1^H NMR. (XLSX 104 kb)

Additional file 3: Table S2. Soybean raw transcript HTSeq counts. (PPTX 467 kb)

Additional file 4: Table S3. RNAseq transcript identification and fold changes of differentially expressed transcripts based on pairwise univariate analyses. (XLSX 4567 kb)

Additional file 5: Table S4. qRT-PCR analysis of randomly selected transcripts for RNAseq dataset validation. (XLSX 106 kb)

Additional file 6: Table S5. Pearson pairwise correlation analysis of metabolites and transcripts. (DOCX 89 kb)

Additional file 7: Table S6. Correlation-scaled loading thresholds used for transcriptomic and metabolomic datasets. (DOCX 41 kb)

Additional file 8: Table S7. Transcript loading coefficients from data integration analysis using O2PLS multivariate analytical methods. (XLSX 65 kb)

Additional file 9: Table S8. Metabolite loading coefficients from data integration analysis using O2PLS multivariate analytical methods. (XLSX 64 kb)

Additional file 10: Table S9. O2PLS transcript variable importance values for transcripts involved in primary metabolism. (XLSX 461 kb)

Additional file 11: Table S10. qRT-PCR primer sequences and thermocycling conditions. (DOCX 21 kb)

Additional file 12: Figure S2. O2PLS score plots comparing different centering and scaling methods. (PPTX 467 kb)

Additional file 13: Figure S3. O2PLS normal probability plots comparing different centering and scaling methods. (PPTX 334 kb)

Additional file 14: Table S11. Summary of O2PLS integration using different scaling and centering methods. (DOCX 60 kb)

## Abbreviations

^1^H NMR: Proton nuclear magnetic resonance
3PGA: 3-phosphoglyceric acid or glycerate-3-phosphate
ADH: Alcohol dehydrogenase
AG: Anastomosis group
AGP: Alpha-glucan phosphorylase
AGT: Alanine-glyoxylate transaminase
Aln: Alanine
AMY: Alpha-amylase
ASN: Asparagine synthetase
BAMY: Beta-amylase
BFF: Beta-fructofuranosidase
BGLUC: Beta-glucosidase
cDNA: Complementary deoxyribonucleic acid
ChlA/B: Chlorophyll A/B binding protein
CoA: Coenzyme A
D_2_O: Deuterium oxide
DE: Differentially expressed
D-gluc: D-glucose
DPSC2: Delta-1-pyrroline-5-carboxylate synthase 2
EtOH: Ethanol
F6P: Fructose-6-phosphate
FDH: Formate dehydrogenase
FDR: False discovery rate
For: Formate
Fruc: Fructose
Fum: Fumarate
G5K: Glutamate-5-kinase
G6P: Glucose-6-phosphate
GABA: Gamma-aminobutyric acid
Gln: Glutamine
Glu: Glutamate
Gluc: Glucose
GO: Gene ontology
GST: Glutathione-S-transferase
h.p.i.: Hours post-inoculation
KEGG: Kyoto encyclopedia of genes and genomes
MHz: Megahertz
MLS: Malate synthase
O2PLS: Bidirectional orthogonal projections to latent structures
PAL: Phenylalanine ammonia lyase
PCA: Principle component analysis
PDA: Potato dextrose agar
PEP: Phosphoenolpyruvate
PEPC: Phosphoenolpyruvate carboxykinase 1
PER: Peroxidase
Phe: Phenylalanine
PLS-DA: Partial least squares-discriminant analysis
PR4: Pathogenesis-related protein 4
PRESS: Prediction error sum of squares
Pro: Proline
PSII: Photosystem II
Q^2^_(cum)_: Cumulated predictive power
QC: Quality control
qRT-PCR: Quantitative real-time polymerase chain reaction
R^2^X, R^2^Y: Fraction of the sum of squares for all X and Y variables, respectively
RFB: Rhizoctonia foliar blight
RNAseq: RNA sequencing
ROS: Reactive oxygen species
Ser: Serine
SRA: Short reads archive
Suc: Succinate
Sucr: Sucrose
TCA: Tricarboxylic acid
THI: Thiamine biosynthesis
Thr: Threonine
Tyr: Tyrosine
UKN2: Hypothetical protein unknown 2
VIP: Variable importance
Wt: Wild type
