# Supplementary material for: An integrated RNAseq-1H NMR metabolomics approach to understand soybean primary metabolism regulation in response to Rhizoctonia foliar blight disease
Source: BMC Plant Biol. 2017 Apr 27;17:84. doi: 10.1186/s12870-017-1020-8 (PMC5408482; doi:10.1186/s12870-017-1020-8)
Supplement: Supplementary file 1 — Representative overlapping 1H NMR spectra of infected soybean leaves and R. solani AG1-IA controls. (PPTX 349 kb) [file 12870_2017_1020_MOESM1_ESM.pptx]

## Slide 1
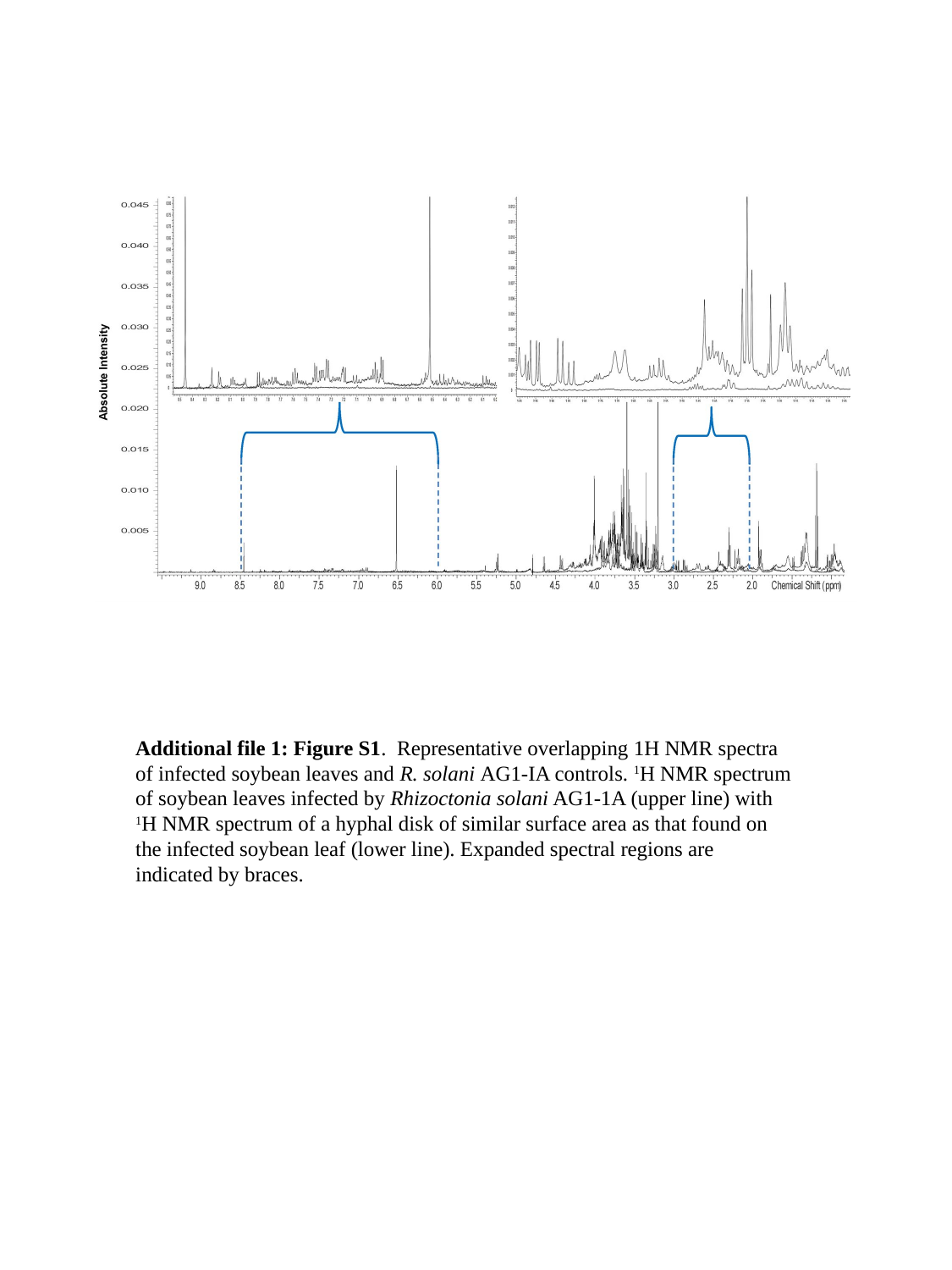

Additional file 1: Figure S1. Representative overlapping 1H NMR spectra of infected soybean leaves and R. solani AG1-IA controls. 1H NMR spectrum of soybean leaves infected by Rhizoctonia solani AG1-1A (upper line) with 1H NMR spectrum of a hyphal disk of similar surface area as that found on the infected soybean leaf (lower line). Expanded spectral regions are indicated by braces.
