# Supplementary material for: An integrated RNAseq-1H NMR metabolomics approach to understand soybean primary metabolism regulation in response to Rhizoctonia foliar blight disease
Source: BMC Plant Biol. 2017 Apr 27;17:84. doi: 10.1186/s12870-017-1020-8 (PMC5408482; doi:10.1186/s12870-017-1020-8)
Supplement: Supplementary file 5 — qRT-PCR analysis of randomly selected transcripts for RNAseq dataset validation. (XLSX 106 kb) [file 12870_2017_1020_MOESM5_ESM.docx]

| **Gene ID** | **Gene annotation^a^** | **qRT-PCR Fold Change ^b^  (*P* value)** | **RNAseq Fold Change ^c^ (*P* value)** |
| --- | --- | --- | --- |
| GLYMA07G15640 | Auxin response factor 19 (ARF19) | 0.758 (0.2361) | 0.168 (0.0010) |
| GLYMA15G07710 | L-DOPA oxidase | 5.258 (0.0315) | INF^d^ (0.0146) |
| GLYMA05G35050 | MYB transcription factor 84 | 4.419 (0.0054) | INF (0.0231) |
| GLYMA03G29950 | Cytochrome P450 93A1 | 19.783 (0.0026) | 14.067 (0.0113) |
| GLYMA08G20230 | Lipoxygenase 10 | 1.972 (0.3933) | 240.54 (0.0004) |
| GLYMA18G07290 | Thiamine C | 0.732 (0.1237) | 0.154 (4.45E-06) |
| GLYMA11G29460 | Dihydroflavonol -4-reductase-like | 1.120 (0.4346) | 3.661 (0.0088) |

**Additional file 5: Table S4**. qRT-PCR analysis of randomly selected transcripts for RNAseq dataset validation.

^a^Gene annotations based on SoyBase database.

^b^qRT-PCR fold changes based on Zhao and Fernald (2005) efficiencies and *P* values based on pairwise comparisons using Student’s *t* test.

^c^RNAseq fold change values based on pairwise comparisons using the negative binomial test and an FDR correction <0.1.

^d^INF represents transcripts that were induced in response to infection, and not detected in control samples.
