# Supplementary material for: An integrated RNAseq-1H NMR metabolomics approach to understand soybean primary metabolism regulation in response to Rhizoctonia foliar blight disease
Source: BMC Plant Biol. 2017 Apr 27;17:84. doi: 10.1186/s12870-017-1020-8 (PMC5408482; doi:10.1186/s12870-017-1020-8)
Supplement: Supplementary file 7 — Correlation-scaled loading thresholds used for transcriptomic and metabolomic datasets. (DOCX 41 kb) [file 12870_2017_1020_MOESM7_ESM.docx]

**Additional file 7: Table S6**. Correlation-scaled loading thresholds used for transcriptomic and metabolomic datasets

|  | **Transcriptomic dataset** | | **Metabolomic dataset** | |
| --- | --- | --- | --- | --- |
| **Latent variable** | 1 | 2 | 1 | 2 |
| **Upper quantile** | 0.024 | 0.020 | 43.0 | 128.6 |
| **Lower quantile** | -0.018 | -0.017 | -38.3 | -79.8 |
