# Supplementary material for: An integrated RNAseq-1H NMR metabolomics approach to understand soybean primary metabolism regulation in response to Rhizoctonia foliar blight disease
Source: BMC Plant Biol. 2017 Apr 27;17:84. doi: 10.1186/s12870-017-1020-8 (PMC5408482; doi:10.1186/s12870-017-1020-8)
Supplement: Supplementary file 11 — qRT-PCR primer sequences and thermocycling conditions. (DOCX 21 kb) [file 12870_2017_1020_MOESM11_ESM.docx]

**Additional file 11: Table S10**. qRT-PCR primer sequences and thermocycling conditions

| Gene Locus | Annotation | Primer name | Primer set (5’-3’) | Product Size (bp) | Annealing temperature (˚C) | Final primer concentration (µM) |
| --- | --- | --- | --- | --- | --- | --- |
| GLYMA04G38210 | Peroxidase 4-like | PER4-F | TGTTACTTGATGGCCCTTCC | 155 | 54 | 0.2 |
|  |  | PER4-R | GTCACGAGCAGCAATGGTAA |  |  |  |
| GLYMA15G07710 | L-DOPA oxidase | PPOA1-F | ATCACATGCAGTGGAAACCA | 151 | 54 | 0.175 |
|  |  | PPOA1-R | AGGCAAAAGGGTTGTTGTTG |  |  |  |
| GLYMA19G36620 | Phenylalanine ammonia lyase 1 | PAL1-F | GTCCAGTACTAAGGGAAGTGATCC | 216 | 54 | 0.15 |
|  |  | PAL1-R | ACTCCTTCTCGGGCAGACTC |  |  |  |
| GLYMA07G15640 | Auxin response factor 19 (ARF19) | ARF19-F | TGCCCGAGGCTAATTCTATC | 245 | 57 | 0.2 |
|  |  | ARF19-R | GGAGCGAGATGGAACATCAT |  |  |  |
| GLYMA03G12240 | Glutamate-5-kinase | G5K-F | ACTCTTGCAAAATGGCCACA | 160 | 54 | 0.175 |
|  |  | G5K-R | ACGCTTCACTTTGGTGACAA |  |  |  |
| GLYMA05G35050 | MYB transcription factor 84 | MYB84-F | TCACATTCACACACCCCTTG | 238 | 54 | 0.2 |
|  |  | MYB84-R2 | TGCTTACATTGTAGCCGTCATATGT |  |  |  |
| GLYMA10G33650 | Glutathione-S-transferase | GST-F | GATGACATGTTTTCTGCAGTTATTG | 174 | 54 | 0.2 |
|  |  | GST-R | CCCAAAAGCTATGTCCATAATGT |  |  |  |
| GLYMA08G20230 | Lipoxygenase 10 | LOX10-F | ATGCAAAAATGTACAAAAACACTCGTA | 188 | 54 | 0.2 |
|  |  | LOX10-R | GGGTGTTCCCAAATCATTGT |  |  |  |
| GLYMA03G29950 | Cytochrome P450 93A1 | 93A1-F | AAGGGCTTAGCCTACGATTCC | 254 | 54 | 0.2 |
|  |  | 93A1-R | AGCGTCATTCTGGATACGATGTT |  |  |  |
| GLYMA18G07290 | Thiamine C | ThiC-F | TCATTCTTGCCTGGGTTTGAT | 148 | 54 | 0.2 |
|  |  | ThiC-R | GCTTCCTTTGTTTGGTTTTGTCT |  |  |  |
| GLYMA11G29460 | Dihydroflavonol -4-reductase- like | DH4R-F | GACTTTGCGAAGGAGACAGG | 162 | 57 | 0.2 |
|  |  | DH4R-R | GTGAGCCGTTCCCATAAAGA |  |  |  |
| GLYMA06G04180 | Hypothetical protein unknown 2 (UKN2) | UKN2-F | GCCTCTGGATACCTGCTCAAG | 79 | 60 | 0.2 |
|  |  | UKN2-R | ACCTCCTCCTCAAACTCCTCTG |  |  |  |
| GLYMA08G45210 | Alpha-glucan phosphorylase | AGP-F | TTGAGCTGGAACAAGCTTACTAT | 279 | 51 | 0.2 |
|  |  | AGP-R | GCCTACCAAGACCACCATTT |  |  |  |
| GLYMA03G04990 | Alanine-glyoxylate transaminase | AGT-F | CTCAAAACTTCCCAGTGATCTC | 156 | 51 | 0.25 |
|  |  | AGT-R | GCCATTGTCCCAGTTGCA |  |  |  |
| GLYMA04G01950 | Alpha-amylase | AMY-F | GTCAGTGGAATCTGGTGGATAC | 238 | 51 | 0.2 |
|  |  | AMY-R | CCAGGTAAGTCACATCCAACTTTA |  |  |  |
| GLYMA02G39320 | Asparagine synthetase | ASN-F | GGTACAATCCTCCTTGGTTCTC | 290 | 51 | 0.2 |
|  |  | ASN-R | GCCTAGATAGTCAGCAACTTCTT |  |  |  |
| GLYMA15G10480 | Beta-amylase | Bamy-F | AGTTCTTCTTGACCTGGTATTC | 199 | 51 | 0.2 |
|  |  | Bamy-R | CGGTATCCGTCTCTATCATTAAG |  |  |  |
| GLYMA05G04290 | Beta-fructofuranosidase | BFF-F | GAACGATCCCAATGGTCCTATG | 248 | 51 | 0.2 |
|  |  | BFF-R | CGTTGGTGGAACCTGTGTATAA |  |  |  |
| GLYMA12G05780 | Beta-glucosidase | Bgluc-F | GACTTCCAGTATGGATGGTTTAT | 247 | 50 | 0.2 |
|  |  | Bgluc-R | CATCACGTACAAATGAGGAATTAG |  |  |  |
| GLYMA01G24530 | Delta 1-pyrroline-5-carboxylate synthase 2 | DPSC2.1-F | AATTTCGTCAGCATCAAACC | 259 | 50 | 0.2 |
|  |  | DPSC2.1-R | CCAATATGACTTCATACCCT |  |  |  |
| GLYMA19G01200 | Formate dehydrogenase | FDH-F | ATGAACTCCTCAGAATCCTTGT | 223 | 50 | 0.2 |
|  |  | FDH-R | GTATTCATCCTAAGTCTATCATAGTAC |  |  |  |
| GLYMA17G13730 | Malate synthase | MLS-F | GAAGATCCAGTGGCTAACGAGGTAGC | 204 | 58 | 0.2 |
|  |  | MLS-R | TTGCTCGGTGATGTTTGCCCCA |  |  |  |
| GLYMA01G23790 | Phosphenolpyruvate carboxykinase 1 | PEPC-F | GGTGAAAGATGAAGTTACTGAGAATG | 251 | 51 | 0.25 |
|  |  | PEPC-R | CTTCGGTAGTTGGTCGAATG |  |  |  |
