# Supplementary material for: An integrated RNAseq-1H NMR metabolomics approach to understand soybean primary metabolism regulation in response to Rhizoctonia foliar blight disease
Source: BMC Plant Biol. 2017 Apr 27;17:84. doi: 10.1186/s12870-017-1020-8 (PMC5408482; doi:10.1186/s12870-017-1020-8)
Supplement: Supplementary file 12 — O2PLS score plots comparing different centering and scaling methods. (PPTX 467 kb) [file 12870_2017_1020_MOESM12_ESM.pptx]

## Slide 1
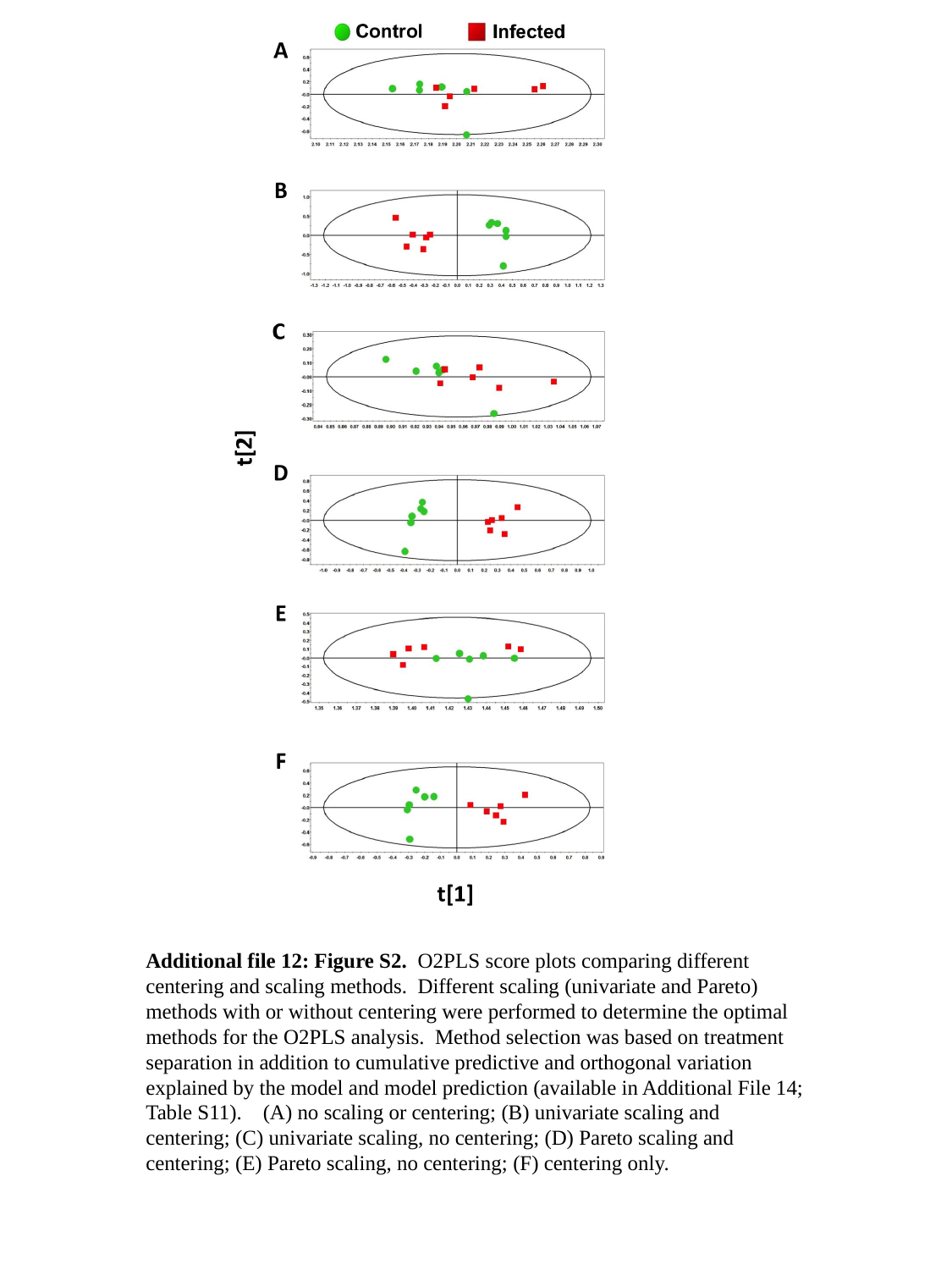

Additional file 12: Figure S2. O2PLS score plots comparing different centering and scaling methods. Different scaling (univariate and Pareto) methods with or without centering were performed to determine the optimal methods for the O2PLS analysis. Method selection was based on treatment separation in addition to cumulative predictive and orthogonal variation explained by the model and model prediction (available in Additional File 14; Table S11). (A) no scaling or centering; (B) univariate scaling and centering; (C) univariate scaling, no centering; (D) Pareto scaling and centering; (E) Pareto scaling, no centering; (F) centering only.
