# Supplementary material for: An integrated RNAseq-1H NMR metabolomics approach to understand soybean primary metabolism regulation in response to Rhizoctonia foliar blight disease
Source: BMC Plant Biol. 2017 Apr 27;17:84. doi: 10.1186/s12870-017-1020-8 (PMC5408482; doi:10.1186/s12870-017-1020-8)
Supplement: Supplementary file 13 — O2PLS normal probability plots comparing different centering and scaling methods. (PPTX 334 kb) [file 12870_2017_1020_MOESM13_ESM.pptx]

## Slide 1
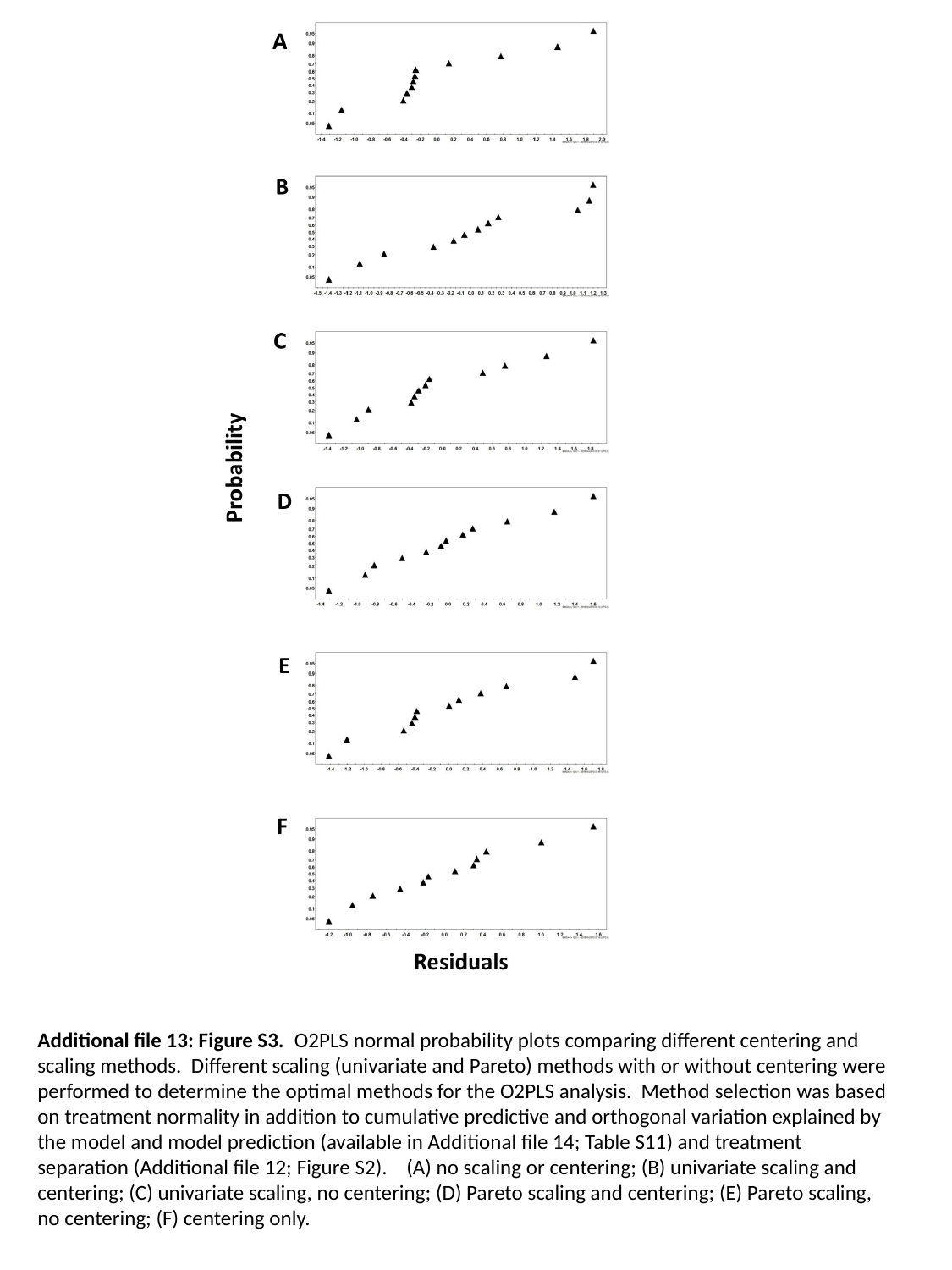

Additional file 13: Figure S3. O2PLS normal probability plots comparing different centering and scaling methods. Different scaling (univariate and Pareto) methods with or without centering were performed to determine the optimal methods for the O2PLS analysis. Method selection was based on treatment normality in addition to cumulative predictive and orthogonal variation explained by the model and model prediction (available in Additional file 14; Table S11) and treatment separation (Additional file 12; Figure S2). (A) no scaling or centering; (B) univariate scaling and centering; (C) univariate scaling, no centering; (D) Pareto scaling and centering; (E) Pareto scaling, no centering; (F) centering only.
