# Supplementary material for: An integrated RNAseq-1H NMR metabolomics approach to understand soybean primary metabolism regulation in response to Rhizoctonia foliar blight disease
Source: BMC Plant Biol. 2017 Apr 27;17:84. doi: 10.1186/s12870-017-1020-8 (PMC5408482; doi:10.1186/s12870-017-1020-8)
Supplement: Supplementary file 14 — Summary of O2PLS integration using different scaling and centering methods. (DOCX 60 kb) [file 12870_2017_1020_MOESM14_ESM.docx]

**Additional file 14: Table S11**. Summary of O2PLS integration using different scaling and centering methods

| Method^#^ | A^$^ | R2X^%^ | R2Y^&^ | Q2^*^ |
| --- | --- | --- | --- | --- |
| CTR | 2+2+0 | 0.529 | 0.909 | 0.613 |
| PAR | 2+1+0 | 0.41 | 0.77 | 0.442 |
| None | 1+1+0 | 0.941 | 0.995 | 0.992 |
| UV | 2+1+0 | 0.396 | 0.645 | 0.271 |
| UVN | 1+1+0 | 0.929 | 0.983 | 0.97 |
| PARN | 1+1+0 | 0.936 | 0.994 | 0.989 |

^#^Scaling and centering methods using for O2PLS integration. CTR, centered with no scaling; PAR, centered and scaled using Pareto variance; None, neither centered nor scaled; UV, centered and scaled using unit variance; UVN, scaled using unit variance, no centering; PARN, scaled using Pareto variance, no centering.

^$^Number of X-Y predictive, X orthogonal and Y orthogonal components for the model.

^%^ The cumulative predictive and orthogonal power explained by the transcripts (X block).

^&^The cumulative predictive and orthogonal power explained by the metabolites (Y block).

^*^The cumulative predictive power of the model.
